# The impact of etiology in lesion-symptom mapping – A direct comparison between tumor and stroke

**DOI:** 10.1016/j.nicl.2022.103305

**Published:** 2022-12-24

**Authors:** E.E. van Grinsven, A.R. Smits, E. van Kessel, M.A.H. Raemaekers, E.H.F. de Haan, I.M.C. Huenges Wajer, V.J. Ruijters, M.E.P. Philippens, J.J.C. Verhoeff, N.F. Ramsey, P.A.J.T. Robe, T.J. Snijders, M.J.E. van Zandvoort

**Affiliations:** a Department of Neurology & Neurosurgery, University Medical Center Utrecht Brain Center, Utrecht University, Utrecht, the Netherlands; b Department of Psychology, University of Amsterdam, Amsterdam, the Netherlands; c St. Hugh's College, Oxford University, UK; d Department of Experimental Psychology and Helmholtz Institute, Utrecht University, the Netherlands; e Department of Radiotherapy, University Medical Center Utrecht, Utrecht, the Netherlands

**Keywords:** Ischemic stroke, Brain tumor, Lesion-symptom mapping, Support vector regression, Cognition

## Abstract

**Introduction:**

Lesion-symptom mapping is a key tool in understanding the relationship between brain structures and behavior. However, the behavioral consequences of lesions from different etiologies may vary because of how they affect brain tissue and how they are distributed. The inclusion of different etiologies would increase the statistical power but has been critically debated. Meanwhile, findings from lesion studies are a valuable resource for clinicians and used across different etiologies. Therefore, the main objective of the present study was to directly compare lesion-symptom maps for memory and language functions from two populations, a tumor versus a stroke population.

**Methods:**

Data from two different studies were combined. Both the brain tumor (N = 196) and stroke (N = 147) patient populations underwent neuropsychological testing and an MRI, pre-operatively for the tumor population and within three months after stroke. For this study, we selected two internationally widely used standardized cognitive tasks, the Rey Auditory Verbal Learning Test and the Verbal Fluency Test. We used a state-of-the-art machine learning-based, multivariate voxel-wise approach to produce lesion-symptom maps for these cognitive tasks for both populations separately and combined.

**Results:**

Our lesion-symptom mapping results for the separate patient populations largely followed the expected neuroanatomical pattern based on previous literature. Substantial differences in lesion distribution hindered direct comparison. Still, in brain areas with adequate coverage in both groups, considerable LSM differences between the two populations were present for both memory and fluency tasks. Post-hoc analyses of these locations confirmed that the cognitive consequences of focal brain damage varied between etiologies.

**Conclusion:**

The differences in the lesion-symptom maps between the stroke and tumor population could partly be explained by differences in lesion volume and topography. Despite these methodological limitations, both the lesion-symptom mapping results and the post-hoc analyses confirmed that etiology matters when investigating the cognitive consequences of lesions with lesion-symptom mapping. Therefore, caution is advised with generalizing lesion-symptom results across etiologies.

## Highlights

• We directly compared LSM in brain tumors and ischemic stroke patients.
• Known neuro-anatomical correlates were found, but differed between etiologies.
• Differences in lesion topography and volume only partly drive divergent findings.
• Lesion-behavior associations are influenced by the etiology causing the lesion.
• Similarities across etiologies were mostly found in white matter tracts.

## 1 Introduction

Topological organization of brain function has been widely studied in patients with brain lesions (Karnath et al., 2018). It remains the most powerful method to infer causality between brain structures and behavior (Catani and Stuss, 2012). The past decades, lesion analyses methods have evolved (Bates et al., 2003), and lesion-symptom mapping (LSM) is currently performed in large samples and is a frequently utilized method in behavioral neurology.

LSM has most frequently been applied in stroke patients, which raises questions about generalizability. The behavioral consequences of lesions from different etiologies, such as brain tumors or stroke, may vary as a result of how they affect the brain (Karnath et al., 2018, Karnath et al., 2004). Firstly, the lesion distribution differs between etiologies, with certain brain areas more likely to be damaged than others. The possible locations for a stroke are confined by the architecture of the vascular system, with a stroke most often occurring in subcortical areas in the territory of the middle cerebral artery. (Sperber and Karnath, 2016, Corbetta et al., 2015, Phan et al., 2005) Primary brain tumors, on the other hand, frequently involve both subcortical and cortical structures in the frontotemporoinsular areas (van Kessel et al., 2019, Kleihues et al., 2007). This nonrandom lesion distribution, which is inherent to each pathology, affects the spatial accuracy of LSM analyses since it limits the possibility to analyze rarely affected brain areas (Ivanova et al., 2021, Mah et al., 2014). First, areas that are rarely damaged are often excluded beforehand from the analyses. Second, for the areas that are included in the analysis, statistical power will vary across the brain and power problems arise when areas are very rarely (or very often) damaged (Kimberg et al., 2007). Moreover, if neighboring or downstream areas are always damaged together, the unique involvement of each voxel to a given behavioral deficit cannot be distinguished.

Furthermore, disease-specific characteristics determine the behavioral consequences. An ischemic stroke is defined by an acute event causing immediate cell death, whereas the rate of brain tumor growth is much slower (months to years, dependent on grade), and neural activity can continue to persist after infiltration by tumor cells (Krainik et al., 2003, Kleihues et al., 2007, Aabedi, 2021). This raises the possibility that the brain recruits mechanisms for neural plasticity in different ways when affected by different pathologies, which in turn may lead to different functional outcomes (Cipolotti et al., 2015, Vaidya et al., 2019).

The use of etiologies other than stroke in lesion-deficit mapping is a highly discussed and controversial topic (Desmurget et al., 2007, Duffau et al., 2003, Plaza et al., 2009, Taphoorn and Klein, 2004). Recently, there has been a rise in lesion studies involving brain tumor patients. The classically theorized neural correlates of behavior, for example the involvement of the posterior perisylvian areas and Broca’s area in language, have mostly been corroborated in this population (Arbula et al., 2020, De Witt Hamer et al., 2013, Habets et al., 2019, Hendriks et al., 2018, Fekonja et al., 2021). In addition, a recent study investigating neuro-anatomical correlates of neglect in a low-grade glioma population found an association between the medial frontal cortex and neglect, which has not yet been documented in stroke lesion studies (Herbet and Duffau, 2022). This underlines the added value of using different populations in lesion-deficit inferences.

Nevertheless, the stroke population remains the predominant research population in LSM studies, while it is still unclear whether etiology-specific biases limit the generalizability of these results. Therefore, we directly compare LSM for memory and language functions from two populations, a tumor versus a stroke population. We expect that both populations will independently show function-specific neural correlates for memory and language functions, as described in previous literature. The aim of this study is to investigate if brain areas where both stroke and tumor populations have adequate coverage, show topographical overlap in lesion-symptom associations. For this study, data from two different studies were combined: a single-center retrospective study in a cohort of treatment-naïve diffuse glioma patients (van Kessel et al., 2019, van Kessel et al., 2022) and a multi-center prospective cohort study in patients with ischemic stroke (Lugtmeijer et al., 2021, Lammers et al., 2022). With a state-of-the-art machine learning-based, multivariate voxel-wise approach, we produced lesion-symptom maps for memory and verbal fluency tasks for both populations separately.

## 2 Methods

### 2.1 Patient recruitment

The University Medical Center Utrecht (UMCU) institutional ethical review board approved both studies in accordance with the Declaration of Helsinki. (World Medical Association, 2013) Detailed in- and exclusion criteria are provided in the Supplementary Methods (see Appendix A).

#### 2.1.1 Tumor patients

The data from the tumor patients was gathered as part of a single-center retrospective study in a cohort of adult treatment-naïve diffuse glioma patients (WHO grade II-IV according to WHO2016 classification (Louis et al., 2016) who underwent awake brain surgery between January 2010 and July 2019 at the (UMCU). As the data of this cohort was previously gathered as part of routine clinical care and was anonymized, informed consent was not required, in agreement with Dutch law. Preoperative neurocognitive assessment and preoperative MRI were part of routine clinical care in preparation for awake craniotomy and used for the current study. Patients who underwent craniotomy under full anesthesia could not be included for the current study, as elaborate preoperative neurocognitive assessment is not part of routine clinical care.

#### 2.1.2 Stroke patients

The data from the stroke patients was gathered as part of the Functional Architecture of the Brain for Vision (FAB4V) study, which is a multi-center prospective cohort study investigating vision and cognition after ischemic stroke. Adult patients with a first-ever symptomatic cerebral ischemic stroke were included in the current study. Written informed consent was obtained from all participants prior to participation. Neurocognitive assessment and an MRI including a T2 FLAIR were performed between three weeks and three months post-infarction. A maximum of one week was allowed between the neurocognitive assessment and the MRI.

### 2.2 Data collection

#### 2.2.1 Neurocognitive assessment

The neuropsychological instruments and corresponding scores for both populations are listed in Supplementary Table 1, along with full test descriptions. The uncorrected scores for each test were transformed into z-scores based on the mean and standard deviation of control populations derived from published norm data. For descriptive purposes, a cognitive impairment was defined as a z-score equal to or lower than −1.5. To assess differences between the tumor and stroke group regarding cognitive performance two types of statistics were performed. Firstly, using Pearson's chi-square tests we investigated whether the relative number of patients with a cognitive impairment was higher in the tumor or stroke group for any of the available cognitive tasks. Secondly, using two-samples t-tests we investigated whether the cognitive performance of the tumor group differed from that of the stroke group for the cognitive tasks that were used in subsequent LSM analyses.

For the LSM analyses, we selected two internationally widely used, standardized psychometric instruments: the Rey Auditory Verbal Learning Test (RAVLT), a verbal learning and memory test that taps into multiple aspects of memory (direct recall, delayed recall, and delayed recognition) and the Verbal Fluency Test. The fluency test is separated into a phonemic fluency (Dutch versions of the Controlled Oral Word Association Test) and semantic fluency (animal) part. The phonemic fluency test is thought to rely more heavily on executive control, while the semantic fluency test is more dependent on correct retrieval of semantic knowledge. The RAVLT and Verbal Fluency Test require both overlapping and distinct cognitive concepts, thereby allowing for specificity in lesion-symptom associations.

#### 2.2.2 Patient characteristics

Patient characteristics for the tumor patients were extracted from the electronic patient file. This data included sex, age at time of surgery, level of education, handedness, and WHO grade. For the stroke patients characteristics were obtained either from the semi-structured interview before the neuropsychological assessment and/or by reviewing the electronic patient file. This data included sex, age, level of education, handedness, stroke location based on MRI, date of stroke onset and medical history.

### 2.3 Image processing

#### 2.3.1 Image acquisition

A T2 FLAIR sequence was used for lesion delineation in the present study. For the glioma patients this was acquired as part of standard clinical care and the pulse-sequence details of the FLAIR MRI varied between glioma patients. T2 FLAIR scans with a slice thickness >5 mm were excluded in order to maintain adequate quality for lesion segmentation for all included glioma patients. The MRI scan that was closest to the pre-operative neurocognitive testing was chosen.

Depending on the medical center, stroke patients underwent a 3T MRI on a Philips Ingenia R5 (Amsterdam UMC and UMCU) or on the Siemens Magnetom Prisma (Radboudumc and UMCG), using a 32-channel head coil. For the Philips scanner the pulse-sequence details were: 3D T2 FLAIR (TI = 1650 ms, TR = 4800 ms, TE = 253 ms, [FOV] = 250 mm, voxel size 1.12 × 1.12 × 0.56 mm). For the Siemens scanner they were 3D T2 FLAIR (TI = 1650 ms, TR = 4800 ms, TE = 484 ms, [FOV] = 280 mm, voxel size 0.9 × 0.9 × 0.9 mm).

#### 2.3.2 Lesion delineation

Both tumor and stroke lesions were segmented on individual T2 FLAIR images. Both tumor and stroke lesion were first drawn in the axial plane and adjusted accordingly in the sagittal and coronal plane. Tumor lesions were delineated using the Smartbrush implemented in the iPlan v3.0 software (BrainLab AG, Feldkirchen, Germany) and represent the total lesion volume, including both tumor and edema. For the tumor patients, a training set (N = 22) was completed in which all tumor regions were drawn by two researchers (EG & VR) under the supervision of an experienced neurologist (TS). After completing the training set, tumor regions were drawn by one of the two researchers. Through consensus meetings with the neurologist (TS), definitive lesion maps were created. The interrater reliability was calculated as the number of voxels included by both raters, in reference to the mean number of voxels selected per rater (Neumann et al., 2009). Based on eight different tumor lesions, the interrater reliability was 93.0 % (range 88.8–96.7 %). Stroke lesions were delineated semi-automatically or manually with the ITK-snap software (Yushkevich et al., 2006). Stroke lesions were delineated by three researchers and in case of doubt for specific scans, a neurologist or radiologist was consulted. The interrater reliability was calculated based on eight stroke lesion masks using the same method as for the tumor interrater reliability. The interrater reliability for all three raters was 81.3 % (range 69.8–91.1 %), as reported previously (Lugtmeijer et al., 2021).

#### 2.3.3 Pre-processing

Each individual’s FLAIR and binary lesion mask (both tumor and stroke) were normalized to an age-specific older adult MNI template using the Clinical Toolbox (Crinion et al., 2007, Rorden et al., 2012) implemented in SPM12 (www.fil.ion.ucl.ac.uk/spm). For unilateral lesions, enantiomorphic normalization was applied to reduce distortions in the normalization due to the lesion (Nachev et al., 2008). For bilateral lesions normalization with cost function, masking was applied (Brett et al., 2001). If the normalization results using enantiomorphic normalization was unsatisfactory for the tumor data, the normalization process was repeated using cost-function masking. The superior lesion mask (defined as visually best representing the original lesion location) was used in subsequent analyses. After spatial normalization, each lesion mask in MNI space was visually compared to the lesion in native space, and manually corrected if needed. Subsequently, the lesion mask was smoothed with a Gaussian kernel of 3 mm at FWHM. For stroke patients, normalization was optimized for patients with enlarged ventricles (>1.5 SD above ventricle volume in elderly template) using a warping regularization reduced by one order of magnitude. The resolution of the normalized lesion maps was 2x2x2 mm^3^. All results are displayed in neurological orientation (left = left hemisphere).

### 2.4 Data analysis

#### 2.4.1 Multivariate lesion-symptom mapping

LSM was applied to test the relationship between lesion status in each voxel and cognitive performance, defined as a Z-score, for each task. For the multivariate LSM we used the support vector regression LSM (SVR-LSM) toolbox running under Matlab2019a (The MathWorks, Inc., Natick, Massachusetts, United States), which is a multivariate regression algorithm based on machine learning (github.com/atdemarco/svrlsmgui) (Zhang et al., 2014, DeMarco and Turkeltaub, 2018). This multivariate method, as opposed to a mass-univariate approach, considers intervoxel correlations and therefore is potentially more sensitive to examine lesion-behavior relationships (Zhang et al., 2014). It has been successfully used and validated in multiple studies including both real and simulated lesion data. e.g. (Karnath et al., 2018, Arbula et al., 2020, Zhang et al., 2014, Zhao et al., 2018). As no clear criteria on parameter choice are available yet, the hyperparameter values were kept at a cost of 30 and a gamma of 5, following the original paper (Zhang et al., 2014). A nonlinear radial basis function kernel was used. A lesion threshold of 3 (i.e. at least 3 patients with lesioned tissue at each voxel) was applied. To test the significance of the beta values, permutation testing was used with 1000 permutations, and a voxelwise threshold of *p* < 0.005 without additional correction for multiple comparisons. Since severity of symptoms is often related to lesion size, we performed lesion volume correction by regressing lesion volume on both behavioral scores and lesion data (Supplementary Table 2), in line with the recommendations by DeMarco and Turkeltaub (DeMarco and Turkeltaub, 2018). To assess the effects of this lesion volume correction, all analyses were repeated without correcting for lesion volume (see Repository for maps). We ran the SVR-LSM for each cognitive task for the tumor and stroke data separately. For each LSM analyses the area containing most voxels with peak significance was calculated. Additionally, we combined the data of both groups and performed the SVR-LSM analyses for each cognitive task using etiology (tumor or stroke) as a covariate on both the behavioral scores and lesion data (see Appendix C). The AALCAT atlas was superimposed on the results to relate significant voxels to brain regions. This atlas combines the 116 regions from the AAL atlas (Tzourio-Mazoyer et al., 2002) with 34 white matter regions from the tractography atlas (Catani and Thiebautdeschotten, 2008). Areas with peak significance and/or areas with at least 10 % of tested voxels significant are reported in the text.

#### 2.4.2 Univariate lesion-symptom mapping

In LSM statistical testing is performed to identify voxels in which individuals with a lesion perform significantly worse compared to individuals without a lesion in that voxel. With the univariate method this statistical test is independently applied to each voxel in the brain, whereas multivariate LSM considers the effect of all lesioned voxels simultaneously. To substantiate the results from the multivariate LSM, additional univariate LSM was performed for each cognitive task for the tumor and stroke data separately, using the statistical analyses software NiiStat (https://github.com/neurolabusc/NiiStat). With continuous behavioral data NiiStat computes statistics using a general linear model. Only voxels damaged in at least 3 patients were considered in the analyses. In line with the multivariate LSM, lesion volume correction was performed. Lesion volume control in NiiStat is based on regressing lesion volume with the behavioral data only. To assess the effects of this lesion volume correction, all analyses were repeated without correcting for lesion volume (see Repository for maps). Permutation testing to correct for multiple testing was set to 10,000 permutations and a voxelwise threshold of *p* <.05 was used. Statistical power maps were generated for each LSM using the “nii_power” function of NiiStat, with a critical one-tailed threshold of *p* < 0.05 and a power of 0.6. Power maps hereby represent the number of patients that would be needed to replicate the results in 60 % of the studies. A maximum number of 200 patients was chosen as adequate power. Next, for each cognitive task the percentage of voxels with adequate power was calculated relative to three different volumes: (1) the voxels included in the LSM (minimum threshold of *3* lesions), (2) the voxels included in the LSM for both the stroke and the tumor group (minimum threshold of 3 lesions in both groups), (3) the total MNI brain volume.

#### 2.4.3 Post-hoc analyses

Post-hoc analyses were performed on multivariate LSM results to directly compare cognitive performance between stroke and tumor in specific areas where the LSM findings in stroke and tumor diverged. Therefore, the interaction effect between lesion status and etiology was tested for specific atlas areas. Atlas areas that showed divergent lesion-symptom associations between stroke and tumor, despite sufficient lesion coverage, were selected for post-hoc analyses. For each subject, we recoded voxel counts per atlas area into damaged (≥5% of voxels affected) and not damaged. Next, areas that were damaged in at least 5 subjects of both the tumor and the stroke sample were selected. Post-hoc analyses were performed per atlas area to directly test whether there was an interaction between lesion status (damaged versus not-damaged) and etiology on cognitive performance. We anticipated that the assumption of normally distributed data would be violated in our dataset and selected a non-parametric alternative. A studentized permutation version of the Wald-type statistic (WTS), as implemented in the GFD R package (Friedrich et al., 2017), was used to test both the main effects of lesion status and etiology as well as their interaction effect. This WTS does not require normally distributed data or variance homogeneity, contrary to the more regular ANOVA statistic.

## 3 Results

### 3.1 Clinical characteristics

**Fig. 1:**
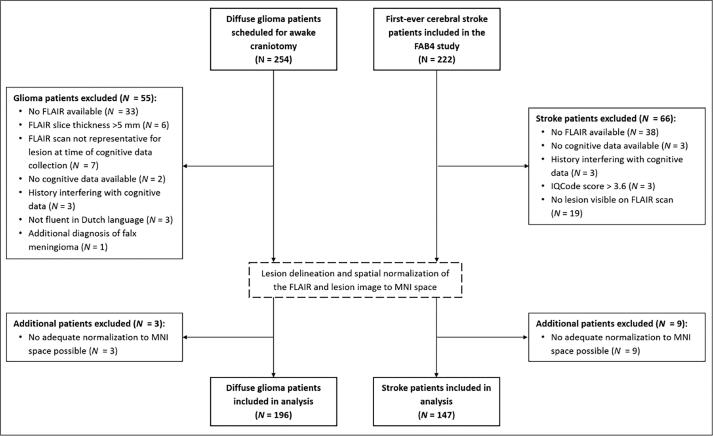
Flow-chart of the in- and exclusion separately for the tumor and stroke population.

In the period between January 2010 and July 2019 254 treatment-naïve diffuse glioma patients (WHO grade II-IV) were scheduled for awake brain surgery and included in the retrospective cohort study. Of these 254 patients, a subset of 196 glioma patients could be included for analyses. Of the 222 first-ever cerebral stroke patients, 147 were included for this analysis (Fig. 1). Average time between ischemic infarct and cognitive assessment was 7.9 weeks (SD = 4.5). Patients from the tumor and stroke group did not differ from each other regarding sex distribution, level of education and hand preference (Table 1). On average, the stroke patients were older, despite a comparable age range. Lesion volume was significantly larger in the tumor group (see Appendix B). While the stroke group had an equal distribution of left and right hemisphere lesions, most tumor patients had a lesion in the left hemisphere. Most tumor patients had a grade IV glioblastoma, IDH-wildtype, followed by grade II + III astrocytoma, IDH-mutant.

**Table 1:** Clinical characteristics for the tumor and stroke patients.

	Tumor	Stroke
N	196	147
Sex (%)		
Male	128 (65.3)	99 (67.3)
Female	68 (34.7)	48 (32.7)
Mean age (SD)	**50.9 (14.1)**	**57.4 (12.9)**
Range	21–81	19–82
Mean educational level (SD)^a^	5.4 (1.3)	5.3 (1.3)
Hand preference (%)^b^		
Left	26 (13.3)	18 (12.2)
Right	163 (83.2)	125 (85.0)
Ambidexter	1 (0.5)	2 (1.4)
Unknown	6 (3.1)	2 (1.4)
WHO 2016 classification (%)		N.A.
II + III astrocytoma IDH-M	55 (28.1)	
II + III astrocytoma IDH-WT	12 (6.1)	
II + III oligodendroglioma IDM-M 1p19q	38 (19.4)	
IV glioblastoma IDH-M	4 (2.0)	
IV glioblastoma IDH-WT	72 (36.7)	
Unknown	15 (7.7)	
Median lesion volume in cm^3^ (SD)	**66.5 (71.9)**	**5.8 (27.1)**
Range	1.2–349.1	0.1–233.2
Lesion location (%)		
Left	**117 (59.7)**	**66 (44.9)**
Right	65 (33.2)	66 (44.9)
Bilateral	14 (7.1)	15 (10.2)

Group differences in clinical characteristics were tested with a Pearson χ2 test or Kruskal-Wallis test when appropriate. Significant difference (*p* < 0.05) are shown in bold.

^a^Educational level was assessed using the Verhage criteria (1964).

^b^Hand preference was self-reported.

### 3.2 Lesion topography

**Fig. 2:**
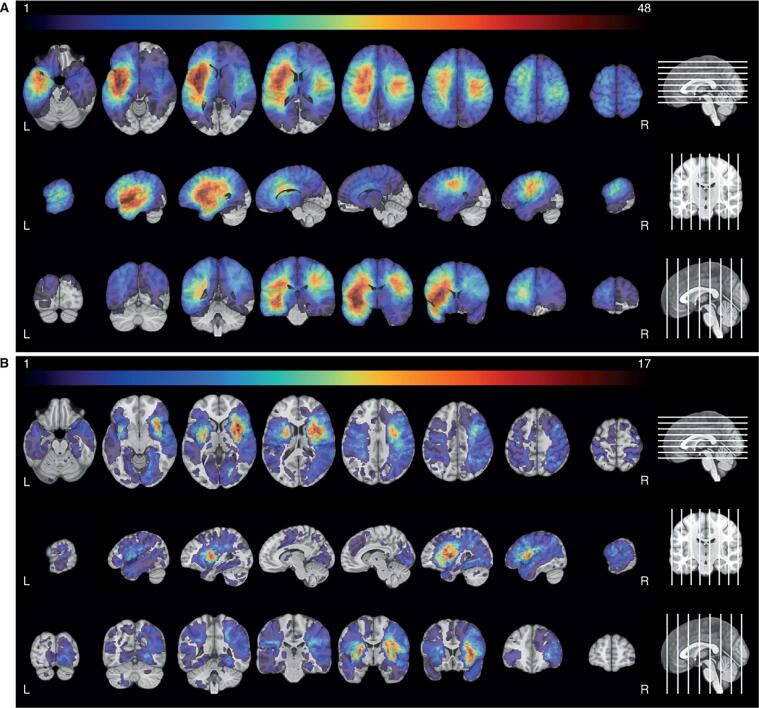
**Lesion prevalence maps for the tumor (A) and stroke group (B) are shown superimposed on the MNI brain.** Multiple slices are shown in the axial, sagittal and coronal plane. The MNI brain on the right indicates the location of the slices shown in the figure. The legend refers to the number of patients with a lesion at that voxel, with red indicating a higher number of patients. The maximum number of patients with an overlap of lesion damage is 48/196 and 17/147 for the tumor and stroke group, respectively. L = left, R = right. (For interpretation of the references to color in this figure legend, the reader is referred to the web version of this article.)

The lesion overlay images of both groups showed a wide distribution of lesions covering both hemispheres (Fig. 2). Tumor lesions were most often located in the left hemisphere, with the highest frequency of lesions in the insula, operculum and superior temporal gyrus. In the right hemisphere the maximum overlap was located more posterior in the pre- and postcentral gyrus. Most stroke lesions were located within the territory of the right middle cerebral artery, with maximum overlap in the insula, putamen and operculum. In total 46.4 % of the voxels were lesioned in at least one patient from both the tumor and stroke group. In 21.8 % of the voxels both the tumor and the stroke group had at least 3 patients with a lesion. See Supplementary Table 4 for an overview of the average percentage of overlap for each atlas structure. For the tumor group, the thresholded lesion overlap map included 125 of the 150 atlas areas. For the stroke group, 98 areas had sufficient coverage. In these lesion maps, the median overlap per area was 12.8 % and 4.8 % for the tumor and stroke group respectively. The number of areas that had >5 % of patients with overlapping lesions in both the tumor and the stroke populations, was limited to 38 out of the 124 atlas areas.

### 3.3 Neurocognitive performance

**Fig. 3:**
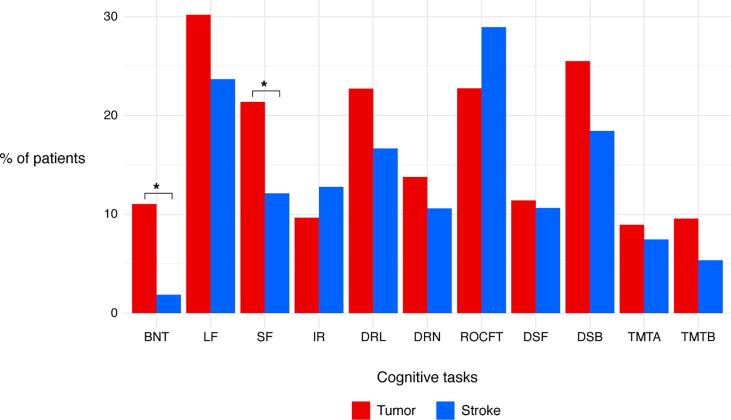
**Percentage of patients with a z-score ≤ -1.5 in the tumor (red) and stroke group (blue) for all available cognitive tasks.** Asterisks indicate significant difference between the tumor and stroke group. Abbreviations: BNT, Boston Naming Task; LF, letter fluency; SF, semantic fluency; IR, immediate recall (RAVLT); DRL, delayed recall (RAVLT); DRN, delayed recognition (RAVLT); ROCFT, Rey Osterreith Complex Figure Test Copy; DSF, digit span forward (WAIS); DSB, digit span backward (WAIS); TMTA, trail making test part A; TMTB, trail making test part B (ratio score). (For interpretation of the references to color in this figure legend, the reader is referred to the web version of this article.)

Fifty-five percent of the tumor patients and 71 % of the stroke patients had a cognitive impairment on at least one cognitive task. Letter fluency (30 %) and working memory (26 %) were the most frequently affected functions in tumor patients, followed by memory recall (23 %), visuoconstructive abilities (23 %), and semantic fluency (21 %). In the stroke group, visuoconstructive abilities (29 %) and letter fluency (24 %) were the most often affected domains. Fewer stroke patients experienced impaired working memory (18 %) and memory recall (17 %) (Fig. 3). Only for naming abilities and semantic fluency, there was a significant difference between stroke and tumor in the number of patients with cognitive impairments (naming: $x^{2}$(1, *N* = 280) = 8.08*p* = 0.004; semantic fluency: $x^{2}$(1, *N* = 277) = 4.20*p* = 0.04). Performance on the continuous measures of the cognitive tasks used for LSM (RAVLT and verbal fluency) was not significantly different between tumor and stroke (immediate recall: *t* = −0.71, *p* = 0.48; delayed recall: *t* = 0.30, *p* = 0.77, delayed recognition: *t* = 0.90, *p* = 0.37, letter fluency: *t* = −0.85, *p* = 0.40, semantic fluency: *t* = −0.12, *p* = 0.90).

### 3.4 Multivariate lesion-symptom mapping results

#### 3.4.1 Direct recall verbal memory (Fig. 4C)

For tumor patients, SVR-LSM analysis indicated that worse performance on the RAVLT Direct Recall was most strongly associated with lesion in the left cingulum (8.0 % of tested voxels in that area were significant). Significant voxels extended into the left hippocampus (60.7 %), parahippocampal gyrus (24.8 %), lingual gyrus (14.2 %) and the fusiform gyrus (9.9 %). Besides lesions in grey matter areas, lesions in the optic radiation (14.2 %) and the inferior longitudinal fasciculus (ILF; 10.4 %) were also significantly associated with worse performance.

For the stroke group, lesions within the putamen were most strongly associated with worse performance (21.0 % of tested voxels significant). Additionally, two left-sided white matter tracts, the inferior fronto-occipital fasciculus (IFOF; 13.7 %) and the uncinate fasciculus (18.5 %) were strongly associated with task performance.

**Fig. 4:**
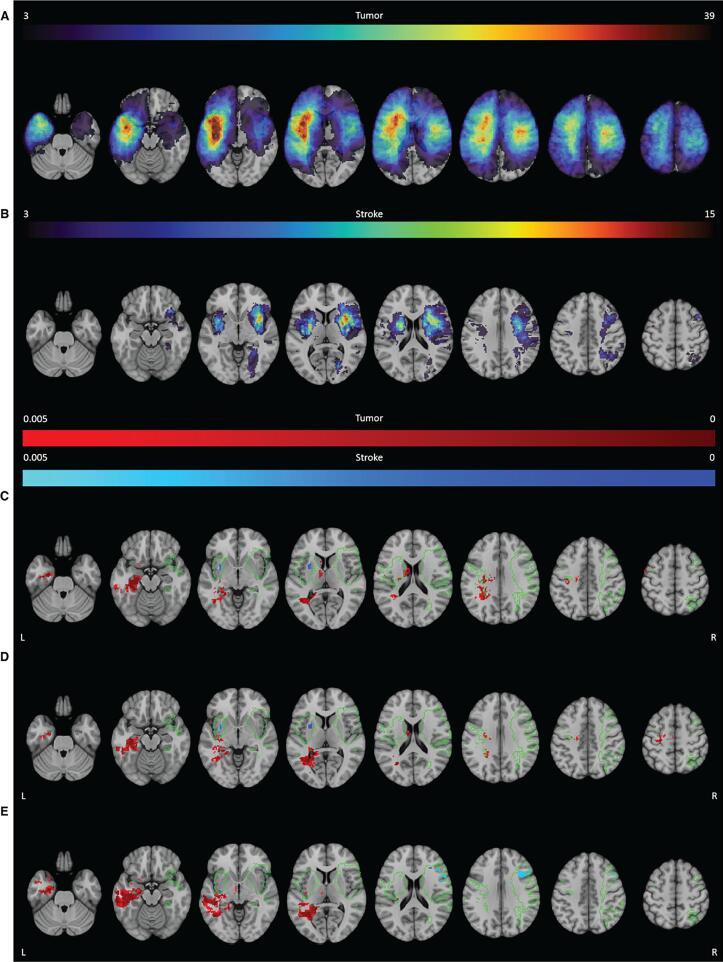
**SVR-LSM results for the RAVLT.** The thresholded lesion overlap for this task is shown for both the tumor (**A**) and stroke group (**B**). The color bar indicates the number of patients with overlapping lesions. Voxels that were significantly associated with worse performance are shown for the tumor group (red) and the stroke group (blue) for the direct recall (**C**), delayed recall (**D**) and delayed recognition (**E**). The colors indicate the p-value for each voxel. The green outline indicates the area within which both groups had a minimum lesion overlap of 3 patients. L = left, R = right. (For interpretation of the references to color in this figure legend, the reader is referred to the web version of this article.)

No directly overlapping significant voxels were found, but in both groups, lesions within the left IFOF were related to worse performance on the direct recall albeit with relatively less significant voxels in the tumor group (Fig. 4**C** and Table 2 for a visual representation of all significant voxels and an overview of the percentage of tested voxels that were significant).

**Table 2:** Detailed descriptions of the anatomical location of significant voxels identified by the SVR-LSM analyses.

Anatomical location	Hem	Direct recall		Delayed recall		Recognition		Letter fluency		Semantic fluency	
		T	S	T	S	T	S	T	S	T	S
**Grey matter regions**											
Precentral gyrus	L			7.2						14.9	**1.1**
Superior frontal gyrus, dorsolateral	R		^a^		^a^		^a^		^a^		^a^
Middle frontal gyrus	R						11.7		^a^		2.8
Inferior frontal gyrus. opercular	L							24.4	^a^	7.1	
Inferior frontal gyrus. opercular	R				1.9		24.8				
Inferior frontal gyrus. triangular	L		^a^		^a^		^a^	12.5	^a^		^a^
Inferior frontal gyrus. triangular	R						4.6				
Rolandic operculum	L							7.4		16.4	
Insula	L							**20.7**		7.7	
Hippocampus	L	60.7	^a^	44.6	^a^	58.5	^a^		^a^		^a^
Parahippocampal gyrus	L	24.8	^a^	15.7	^a^	19.7	^a^		^a^		^a^
Amygdala	L		^a^		^a^	6.6	^a^		^a^		^a^
Calcarine fissure	L		^a^	6.2	^a^		^a^		^a^		^a^
Lingual gyrus	L	14.2	^a^	8.2	^a^	5.4	^a^		^a^		^a^
Fusiform gyrus	L	10.0	^a^	10.0	^a^	14.4	^a^		^a^		^a^
Caudate nucleus	L	9.9									
Putamen	L		**21.0**		**18.7**				16.5		
Thalamus	L	7.2	^a^		^a^	6.0	^a^		^a^		^a^
Heschl gyrus	L									15.4	
Middle temporal gyrus	L		^a^		^a^	**24.6**	^a^		^a^		^a^
Inferior temporal gyrus	L		^a^	10.6	^a^	17.4	^a^		^a^		^a^

**White matter regions**											
Anterior Segment	L									31.3	
Arcuate Fasciculus	L	6.7								**16.0**	
Cingulum	L	**8.0**	^a^	7.0	^a^	5.9	^a^		^a^		^a^
Cortico-ponto-cerebellar tract	L	8.4	^a^		^a^	9.4	^a^		^a^	5.6	^a^
Corticospinal tract	L	8.5							**9.6**	14.9	3.2
Forni	L	7.7	^a^	6.3	^a^	6.2	^a^		^a^		^a^
Inferior Longitudinal Fasciculus	L	10.4	^a^	**19.6**	^a^	40.3	^a^		^a^		^a^
Inferior Fronto-Occipital Fasciculus	L	7.3	13.7	12.4	7.4	14.1	4.8	8.1			
Internal Capsule	L	5.4		5.5		6.2			8.6	7.8	
Long Segment	L							9.0		32.9	
Optic Radiation	L	14.2		19.3		23.6	**39.0**		^a^		
Posterior Segment	L	8.6	^a^	9.2	^a^	20.8	^a^		^a^		^a^
Uncinate Fasciculus	L		18.5		7.2		6.4		^a^		

Abbreviations: Hem, hemisphere; L, left; R, right; T, tumor; S, stroke.

Numbers represent the percentage of significant voxels proportioned to the total of tested voxels for that atlas area in SVR-LSM analyses. Bold values contain the peak z-values in most voxels for each cognitive task. If a task was found to be associated with >5 atlas areas, only tested areas containing at least 5% significant voxels are reported. Smaller clusters are included if they contained peak z-values for that task.

^a^Regions with <5% coverage of that atlas area in that population.

#### 3.4.2 Delayed recall verbal memory (Fig. 4D)

The SVR-LSM analyses in tumor patients identified a cluster of voxels located within the left ILF (19.6 %) that was associated with worse performance on the RAVLT delayed recall. Lesions within the left hippocampus (44.6 %), optic radiation (19.3 %), parahippocampal gyrus (15.7 %), IFOF (12.4 %), inferior temporal gyrus (10.6 %) and fusiform gyrus (10.0 %) were also associated with worse performance.

For stroke patients, lesions within the left putamen (18.7 %) were associated most with task performance. For both patient groups the IFOF was associated with task performance, but there were no directly overlapping significant voxels (Fig. 4**D** and Table 2). Moreover, the relative number of significant voxels was higher in the tumor group than in the stroke group for this area.

#### 3.4.3 Delayed recognition verbal memory (Fig. 4E)

Tumor lesions within the left middle temporal gyrus (24.6 %) were strongly associated with worse performance on the RAVLT delayed recognition. Significant voxels were also found in other grey and white matter areas in the left hemisphere, including the hippocampus (58.5 %), ILF (40.3 %), optic radiation (23.5 %), posterior segment of the arcuate fasciculus (20.8 %), parahippocampal gyrus (19.7 %), inferior temporal gyrus (17.4 %), fusiform gyrus (14.4 %) and the IFOF (14.1 %).

For stroke patients not only lesions in the left optic radiation (31.1 %), but also in the inferior frontal gyrus opercular (24.8 %) and middle frontal gyrus (11.7 %) in the right hemisphere were associated with recognition performance. Results from both etiologies indicated the left optic radiation to be involved in task performance. The left IFOF was also found in both groups, albeit with a lower number of significant voxels in the stroke group (Fig. 4**E** and Table 2).

#### 3.4.4 Letter fluency (Fig. 5)

The SVR-LSM analyses indicated that tumor lesions within the insula (20.7 %) were highly associated with worse letter fluency performance. Significant voxels extended into grey matter in the left inferior frontal gyrus opercular (24.4 %) and triangular (12.5 %).

**Fig. 5:**
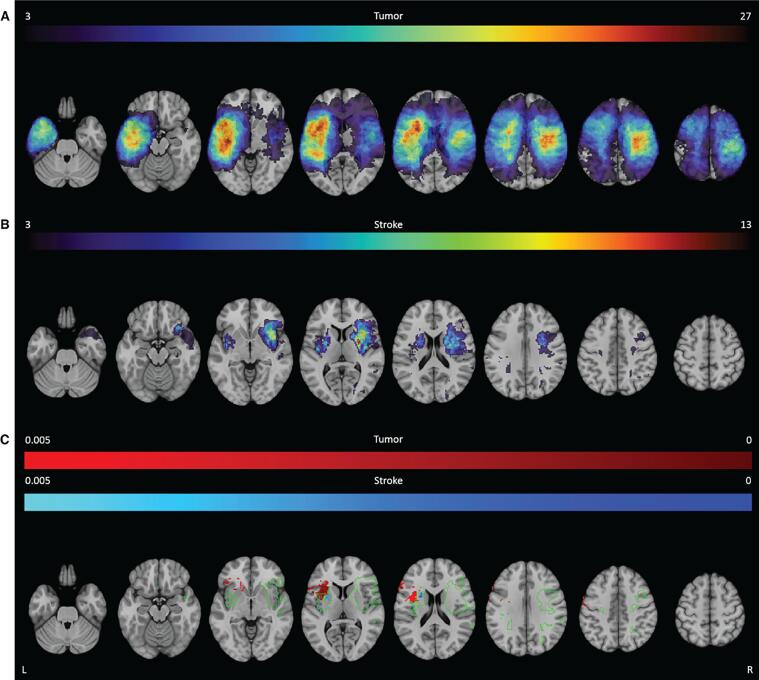
**SVR-LSM results for the letter fluency.** The thresholded lesion overlap for this task is shown for both the tumor (**A**) and stroke group (**B**). The color bar indicates the number of patients with overlapping lesions. Voxels that were significantly associated with worse performance are shown for the tumor group (red) and the stroke group (blue) for the letter fluency (**C**). The colors indicate the p-value for each voxel. The green outline indicates the area within which both groups had a minimum lesion overlap of 3 patients. L = left, R = right. (For interpretation of the references to color in this figure legend, the reader is referred to the web version of this article.)

Stroke lesions within the left corticospinal tract (9.6 %) and putamen (16.5 %) were most associated with the task performance. No overlapping region between the tumor and stroke group was found associated with letter fluency performance (Fig. 5 and Table 2**)**.

#### 3.4.5 Semantic fluency (Fig. 6)

Performance on a semantic fluency task was strongly associated with tumor lesions in the left arcuate fasciculus (16.0 %). Additionally, other involved white matter areas included the left long segment of the arcuate fasciculus (32.9 %), anterior segment of the arcuate fasciculus (31.3 %) and the corticospinal tract (14.9 %). Additionally, lesions in the grey matter of the rolandic operculum (16.4 %), Heschl’s gyrus (15.4 %) and the precentral gyrus (14.9 %) were associated with worse task performance.

**Fig. 6:**
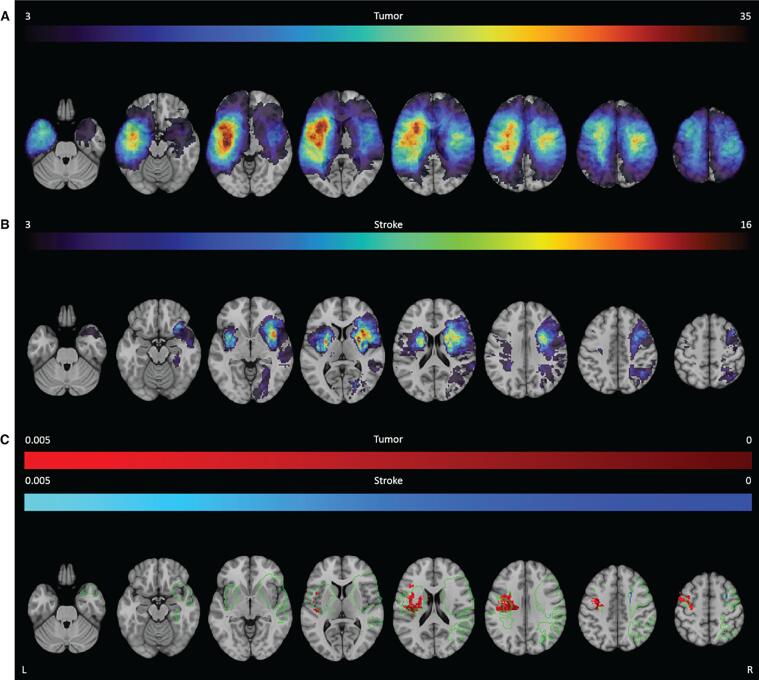
**SVR-LSM for the semantic fluency.** The thresholded lesion overlap for this task is shown for both the tumor (**A**) and stroke group (**B**). The color bar indicates the number of patients with overlapping lesions. Voxels that were significantly associated with worse performance are shown for the tumor group (red) and the stroke group (blue) for the semantic fluency (**C**). The colors indicate the p-value for each voxel. The green outline indicates the area within which both groups had a minimum lesion overlap of 3 patients. L = left, R = right. (For interpretation of the references to color in this figure legend, the reader is referred to the web version of this article.)

Stroke lesions within the left precentral gyrus (1.1 %) were most associated with worse semantic fluency scores. In general, most brain areas did not overlap between the tumor and stroke LSM (Fig. 6 and Table 2**)**. Nevertheless, lesions in the left corticospinal tract and left precentral gyrus were associated with the task performance in both groups. Of note is that the percentage of significant voxels was lower in the stroke group, even though they contained peak significant values.

#### 3.4.6 Specificity of lesion-symptom results

Specificity of the lesion-symptom results was assessed by calculating the amount of overlap between voxels that were significantly related to any of the three memory tasks compared to the two fluency tasks. For the tumor group, there was a 2.0 % overlap between voxels related to both memory and fluency performance. This overlap was mainly located in and around the left cortico-spinal tract, internal capsule, arcuate fasciculus and corpus callosum. Additionally, some overlapping voxels were found in the left precentral gyrus. 13.9 % of voxels associated with all three memory tasks and 33.9 % with any two memory tasks. Overlapping areas for all three tasks were located in the left hippocampus, parahippocampal gyrus, the cingulum, corpus callosum and ILF. For any two memory tasks this extended into the left fusiform gyrus, the temporal gyrus and optic radiations. Between the two different fluency tasks, there was an 8.6 % overlap in significant voxels. These were located in and around the left insula, inferior frontal gyrus, rolandic operculum and arcuate fasciculus.

For the lesion-symptom results in the stroke group there was a 4.9 % overlap between voxels related to both memory and fluency task performance. Overlapping voxels were mainly located in the left putamen and the cortico-spinal tract. 1.3 % of voxels were associated with all three memory tasks and 18.4 % with any two memory tasks. For all three memory tasks the overlapping voxels were mainly located in the left IFOF and optic radiations. For any two memory tasks this extended into the left putamen and uncinate fasciculus and the right insula and IFOF. There were no voxels significantly related to both the phonological and semantic fluency task.

#### 3.4.7 Post-hoc analyses (Fig. 7)

**Fig. 7:**
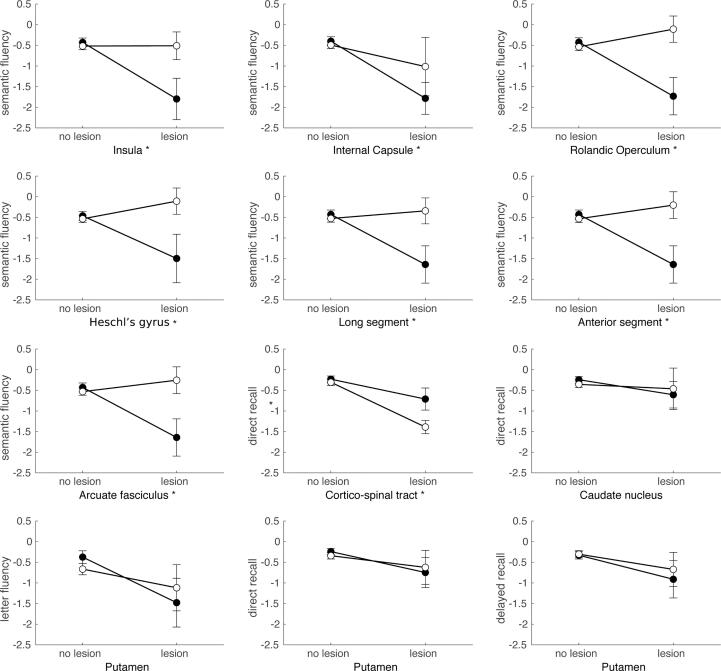
**Post-hoc interaction plots based on SVR-LSM results.** Plots show the mean and standard error of the cognitive performance per etiology (tumor [black] versus stroke [white]), and lesion status (lesion versus no lesion). All brain areas were in the left hemisphere and their labels are depicted below the individual plots. Cognitive performance on the different tasks (semantic fluency, letter fluency, direct recall RAVLT and delayed recall RAVLT) is shown as z-scores on the y-axis. An asterisk next to the label indicates a brain area for which a significant interaction effect was found.

Post-hoc interaction analyses were performed to further examine atlas areas that showed lesion-symptom associations for only one of the two etiologies, despite sufficient lesion coverage in both. A complete overview of the post-hoc results can be found in Fig. 7.

For the lesion-symptom associations found in the tumor group only, post-hoc analyses using a permutation version of the Wald-type statistic (WTS) revealed significant interaction effects between lesion status (damaged versus not damaged) and etiology (stroke versus tumor) in 6 out of 9 tested atlas areas. Interaction effects were found for semantic fluency performance in the left insula (*W*_T_ = 5.08, *p* = 0.033), left rolandic operculum (*W*_T_ = 9.40, *p* = 0.01), left Heschl’s gyrus (*W*_T_ = 4.69, *p* = 0.048) and white matter areas of the left arcuate fasciculus (*W*_T_ = 6.88, *p* = 0.016) (including the long segment (*W*_T_ = 7.39, *p* = 0.015) and anterior segment (*W*_T_ = 6.21, *p* = 0.023)), with worse performance for tumor lesions. For the association between the left internal capsule and semantic fluency there was only a significant main effect of lesion status (*W*_T_ = 5.43, *p* = 0.04). This association was more pronounced in the tumor group, but a similar trend was present in the stroke group. The association between the left corticospinal tract and delayed recall performance was even stronger in stroke than in tumor, leading to a significant main effect of lesion (*W*_T_ = 22.6, *p* ≤ 0.001) as well as a significant main effect of etiology (*W*_T_ = 4.88, *p* = 0.045). The association between the left caudate and worse direct recall performance was not confirmed in post-hoc analysis.

For the lesion-symptom associations found solely in the stroke group, only the left putamen had sufficient lesion coverage to allow further post-hoc analysis. None of the post-hoc analysis for the left putamen, showed significant main or interaction effects. The variability in cognitive performance was high in the subset of subjects with left putamen lesions, independent of etiology.

### 3.5 Univariate lesion-symptom mapping results

We performed univariate LSM with lesion volume control to corroborate results found in the multivariate LSM analyses. For the tumor population, worse performance on the RAVLT Direct Recall and Delayed Recognition was not related to specific lesion locations when using univariate analyses. For the RAVLT Delayed Recall, letter fluency and semantic fluency voxels related to worse performance on the univariate analyses largely overlapped with those from the multivariate analyses (Supplementary Figures 7 and 8). Regions indicated by multivariate analyses seem to expand from the overlapping regions and encompass larger brain areas.

For the stroke patients, univariate LSM did not indicate a significant relationship between lesion location and cognitive performance for the RAVLT direct recall, delayed recall or delayed recognition. For the two fluency tasks (letter and semantic) voxels related to worse performance on the univariate analyses largely overlapped with those from the multivariate analyses (Supplementary Figures 9 and 10). Regions indicated by multivariate analyses seem to expand from the overlapping regions and encompass larger brain areas. As less voxels were significant in the univariate analyses for both the tumor and stroke group, the minimal overlap that was found with multivariate LSM decreased even further when using univariate LSM results.

#### 3.5.1 Statistical power

To assess possible difference in power throughout the brain, statistical power maps were created for each univariate LSM for the tumor and stroke group separately (Supplementary Figure 11). Adequate power was set at a maximum of 200 patients needed to replicate the results in 60 % of the studies. The relative number of voxels with an adequate power was calculated relative to (1) the coverage for the specific cognitive task (minimum threshold of 3 lesions), (2) the overlap in coverage (minimum threshold of 3 lesions in both the stroke and tumor group), and (3) the total MNI brain volume. For each cognitive task, more voxels had adequate power in the tumor than in the stroke group (Table 3).

**Table 3:** Percentage of voxels with adequate power for univariate LSM (maximum of 200 patients needed to replicate results in 60% of studies) relative to the etiology-specific coverage for the cognitive task, the overlap in coverage between the tumor and stroke group for the cognitive task and the complete MNI volume.

Cognitive task	Coverage		Overlap		Whole-brain	
	Tumor	Stroke	Tumor	Stroke	Tumor	Stroke
Direct recall	31.8	18.2	28.9	19.4	23.7	2.0
Delayed recall	32.2	13.0	26.8	13.7	24.0	1.4
Delayed recognition	20.8	10.5	20.4	11.2	15.5	1.1
Letter fluency	42.6	34.4	30.9	27.7	26.6	2.1
Semantic fluency	42.9	16.0	30.0	17.0	30.2	2.4

### 3.6 Lesion volume correction

Both multivariate and univariate LSM were repeated without correcting for lesion volume (maps available in **Repository**). All LSM results were visually compared and no major differences between lesion volume corrected and uncorrected results were found. Corrected LSM tended to encompass smaller, but overlapping, areas with the uncorrected LSM. From the non-corrected LSM, voxels expanded from those regions, thereby indicating more voxels were significantly related to task performance when lesion volume was not taken into account. Largest differences between corrected and uncorrected LSM results were seen for the tumor group, especially for the RAVLT Delayed recognition and Semantic fluency task.

## 4 Discussion

In this study, we investigated whether etiology-specific biases impact brain-behavior associations in LSM. This study is unique in comparing results from state-of-the-art machine learning-based, multivariate LSM between two large study-populations, a tumor versus a stroke population. We expected overlapping lesion-symptom associations in brain areas affected in both populations and population-specific lesion-symptom associations in brain areas covered per pathology. Despite our large sample sizes, substantial differences in lesion distribution conditioned the degree of lesion overlap between stroke and tumor and hindered direct comparison for most brain areas. Still, both our LSM results as well as the post-hoc analyses suggested that the cognitive effects of damage in certain brain areas depend on the underlying pathology.

### 4.1 Lesion topography

Both study samples showed a wide distribution of lesions covering both hemispheres. Tumors were most prevalent in the left hemisphere, with the highest frequency in the insula, operculum and superior temporal gyrus, while strokes were predominantly located within the territory of the right middle cerebral artery. As patients selected for awake surgery are characterized by tumor localization in eloquent areas, such as language and motor areas, this left-sided predominance was to be expected. Nevertheless, lesion coverage was adequate in both hemispheres. Overall, lesion overlap was both more widespread and higher per brain structure in the tumor group. Lesion volume was skewed to larger volumes in the tumor group, and to smaller lesions for stroke. These differences in lesion distribution, overlap and volume had repercussions for the statistical power throughout the brain and consequently the ability to compare LSM; only one-fifth of voxels survived the minimal power threshold for direct comparison.

### 4.2 Lesion-symptom mapping

In both samples cognitive impairments were present in more than half of the patients. Despite the clear differences in lesion distribution between tumor and stroke, the cognitive profile was quite similar on a group-level. Importantly, there were no significant group-level differences in performance on the memory and fluency tasks. Lesion volume was correlated with cognitive performance in the tumor group. However, the larger lesion volume in the tumor group did not result in more cognitive impairments compared to the stroke population. Memory and fluency tasks and their neuroanatomical correlates present differentiated cognitive processes, as expected. That is, more resemblance was found in critical neuroanatomical locations within than between cognitive domains. For both populations the overlap between the tasks was mainly located in white matter pathways which have been independently related to memory and fluency performance previously (Habets et al., 2019, Pisoni et al., 2019, Na et al., 2021, Dick et al., 2014, Fridriksson et al., 2013, Thye et al., 2021). As these white matter tracts have also been related to other cognitive processes, like attention, processing speed and executive functioning (Habets et al., 2019, Zhao et al., 2018, Bettcher et al., 2016), it is most likely that these overlapping regions are involved in a wide range of cognitive processes, including memory and fluency. Of note, overlapping lesions for the tumor and stroke group were mostly found in the white matter and subcortical areas. Thereby the power to find overlapping LSM results between our groups was highest in these white matter areas. In addition, lesion volume correction was performed to eliminate the effect of differences in lesion size between the groups on LSM results as much as possible. In the uncorrected LSM we mainly found larger areas associated with task performance for the tumor group, with significant voxels expanding from the regions found with the corrected LSM. Nevertheless, the overlap between the tumor and stroke LSM results only changed slightly for the uncorrected LSM results, indicating lesion volume control was not a great influence in our comparison between etiologies.

Regions associated with memory performance in the tumor group were in both the grey and white matter areas surrounding the left hippocampus. These critical memory-regions are known from literature as part of the core network for episodic memory postulated by Benoit and Schachter (Benoit and Schacter, 2015) as well as the human memory circuit from Ferguson and colleagues (Ferguson et al., 2019). The left hippocampal areas had insufficient coverage in the stroke group. The majority of memory-regions found in either sample have been identified or are adjacent to regions found previously in tumor and stroke populations (Habets et al., 2019, Pisoni et al., 2019, Biesbroek et al., 2015).

The location of the fluency-related voxels, mainly in the left temporal and frontal lobe, aligns well with previous knowledge of the language system, and fluency in particular (Fekonja et al., 2021, Na et al., 2021). Regions critical for letter fluency performance are located mostly in sensorimotor regions, while regions located around Broca’s area were critical for semantic fluency. This distinction in neuroanatomical fluency correlates was also found in a large-scale study in 1231 S patients (Biesbroek et al., 2021). Similar to our results, they found lesion-symptom associations between frontotemporal regions and letter fluency, while more posterior temporal regions were crucial for semantic fluency. The importance of white matter to language functions in tumour patients was also found in a recent LSM study in patients suffering from left perisylvian gliomas (Fekonja et al., 2021). So, our study shows function-specific neural correlates for the memory and language functions that are corroborated by prior literature, supporting the validity of our LSM results in both populations.

That said, the LSM presents large differences between tumor and stroke. This can partly be explained by the etiology-specific brain coverage that allows for investigation of distinct brain areas in tumor versus stroke. However, also in brain areas affected in both groups the overlap was minimal; we found no directly overlapping voxels between the LSM for tumor and stroke on any task. The similarities we did find mostly involved associated white matter tracts, in accordance with previous literature (Habets et al., 2019, Pisoni et al., 2019, Biesbroek et al., 2021, Li et al., 2017). For example, verbal memory tasks were associated with the left IFOF in both populations. Additionally, the left corticospinal tract and precentral gyrus were associated with semantic fluency performance for both populations. These results were confirmed in the combined LSM analyses in which the relation between lesion location and cognitive performance, irrespective of etiology, was tested. Differences did exist, however, between these combined and separate etiology-specific analyses with some brain areas being critical areas in only one of the two analyses. Again, correction for lesion etiology in the combined analyses was only possible in regions where both populations had adequate lesion coverage. Moreover, as was also shown by the statistical power of the univariate LSM, more voxels had adequate power in the tumor group. Thereby, the results of the combined analyses are likely mainly driven by the lesion data available in the tumor population, due to the uneven sample distribution at the voxel level.

Though power inequalities could explain some of the differences, post-hoc analyses in regions with adequate coverage in both groups confirmed that the results of lesion-deficit inference strongly differ. To illustrate, in several brain areas the negative impact of a lesion in this area on semantic fluency performance was only present when this brain area was lesioned by a tumor. This was, however, not the case for all investigated brain areas. For example, all post-hoc analyses in brain regions that were significantly associated with cognitive performance in the stroke group, did not show a similar etiology interaction effect. Here, differences might be better explained by the large variation in cognitive performance, thereby failing to reach significance in the lesion-symptom mapping analyses.

We specifically investigated the effect of etiology on lesion-symptom associations identified by lesion-symptom mapping. In both tumor and stroke, however, the impact of a lesion can extend far beyond local changes in circumscribed tissue visible on standard imaging techniques, and a small lesion can have widespread effects on behavior (van Kessel et al., 2019, van Kessel et al., 2022, Bowren et al., 2022, Thiebaut de Schotten and Forkel, 2022, Carrera and Tononi, 2014, Siegel et al., 2022, Silvestri et al., 2022). This might also explain the similarity in cognitive profiles between the two samples despite clear differences in lesion location and volume. It is likely that the degree of overlap between both samples is underestimated in terms of structural and functional connections. Nevertheless, this does not explain why we found different cognitive effects in brain areas with overlapping lesion coverage.

### 4.3 Etiology-specific cognitive effects

Few studies discuss the impact of etiology on lesion-behavior associations. Anderson and colleagues matched a small group of brain tumor patients (*n* = 17) to patients with unilateral stroke based on lesion location and compared cognitive outcome (Anderson et al., 1990). They showed substantial differences in cognitive performance, with tumor patients outperforming matched stroke subjects and showing relatively mild impairments. Accordingly, it is still often assumed that the location of a tumor bears little explanatory value. This assumption was challenged by Shallice and colleagues (Shallice et al., 2010), who did find selective and dissociated visuo-spatial deficits in a series of brain tumor patients.

Two studies of more recent date directly compared the impact of different etiologies on the cognitive sequelae of lesions. Frontal-based functions were compared between tumor and stroke patients and showed no differences in performance (Cipolotti et al., 2015). Here, the authors selected patients with frontal lesions, but did not perform further LSM. In a LSM study of apraxia, tumor and stroke patients were included and analyzed both separately and combined (Manuel et al., 2013). Critical brain areas for apraxia matched previous literature, but differed in the tumor and stroke group. This is in line with our findings, since we could relate eloquent functions to areas with an established role in that function.

#### 4.3.1 Tumor patients in lesion-symptom mapping

Different factors could explain the minimal overlap in critical areas, even in brain areas with adequate lesion coverage in both groups. Some factors are inherent to lesion studies in general, others are etiology specific. First, both tumor and stroke have a systematic bias in lesion anatomy. For example, if region A and region B get blood supply from the same artery, a stroke in this artery will likely damage both regions. Suppose that only region A is causally related to the function under investigation, a lesion-symptom association for region B can be found as a mere side-effect (Mah et al., 2014, Sperber, 2020, Lorca-Puls et al., 2018). The possibility of collateral damage is higher when lesion size increases, irrespective of etiology. Thus, it is possible that some regions are associated with the behavioral deficit in one population but not the other, because of correlated damage occurring in one population only. Hence, the lesion-symptom association in question (region B) is not a causal relation.

Second, several tumor-related factors could cause over- or underestimation of affected tissue. These include the possibility of functional reorganization, tumor invasion of apparently healthy tissue, and vice versa the possibility of functional tissue within the area demarked by abnormal MRI signal. Both temporal and spatial differences in activity have been observed in glioma-infiltrated cortex compared to healthy cortex during language tasks (Aabedi, 2021). This could indicate that glioma-infiltrated cortex also negatively impacts cognitive performance. Since our results indicated that on group level larger tumor lesions did not lead to worse cognitive performance compared to the smaller stroke lesions, this increases the likelihood of functional reorganization in our tumor group. However, in the current tumor sample a large proportion of patients had high-grade gliomas (38.7 %), while the chance of functional reorganization or function preservation within the tumor area is highest in slow-growing lesions, like low-grade glioma (Desmurget et al., 2007). Last, mass effect, shift and infiltration of white matter pathways, and increased intracranial pressure may cause noise in lesion-symptom associations. These effects do not always lead to destruction of structures, but impact lesion-symptom associations through spatial displacements that are specific for brain tumors, and may change over time (van Kessel et al., 2020). These tumor-related factors have been used as arguments against the use of tumor series in function localization. On the other hand, our study also clearly illustrates the advantage of using multiple etiologies. Firstly, the ability to investigate a broader range of brain structures unconstrained by the nonrandom lesion distribution inherent to each pathology. Secondly, LSM in tumor series may be especially valuable to make assumptions about function localization prior to neurosurgery.

### 4.4 Study limitations and further directions

#### 4.4.1 Methodological choices

LSM tools are used to identify the neural structures critical for a given behavior. However, multiple methodological choices can influence subsequent LSM results. In the current study we performed LSM using two different types of analyses; a univariate and multivariate approach. Previous research has indicated neither method is superior, but rather both have advantages and disadvantages (Karnath et al., 2018, Ivanova et al., 2021). For example, the multivariate SVR-LSM used in the current study could have been affected by the hyperparameter selection. As no clear criteria on parameter choice are available yet hyperparameter values were kept in line with the original paper. However, this selection was based on a specific stroke sample, with larger lesion sizes (median 76.79 cc) than our current sample (Zhang et al., 2014). Therefore, we corroborated our multivariate LSM results using univariate LSM. However, univariate voxelwise LSM can be conservative due to strict multiple testing corrections. This was also observed in our results with only a small number of voxels surviving significance testing. Nonetheless, most regions related to task performance in the univariate LSM overlapped with those from the multivariate LSM, thereby corroborating our results. Still, we cannot directly compare results from the univariate and multivariate analyses, since the lesion volume correction differed between methods and multiple comparison corrections were only performed for univariate analyses.

Additionally, we chose to perform lesion volume correction in order to eliminate the effect of lesion volume on our comparative analyses as much as possible. The importance of incorporating lesion volume into LSM analyses has been recommended for several years for both multivariate and univariate LSM (DeMarco and Turkeltaub, 2018, Sperber and Karnath, 2017, de Haan and Karnath, 2018). Nevertheless, for the SVR-LSM this means a double covariate control was performed, thereby possibly further decreasing the power to find overlapping regions between the tumor and stroke group. When comparing uncorrected and corrected LSM results, more areas were related to worse cognitive performance when lesion volume was not taken as a covariate, for both univariate and multivariate analyses. As to be expected, this effect was most pronounced for the tumor population as lesion size was significantly related to task performance (memory and fluency) in the tumor, but not stroke group. However, regardless of the specific LSM method that we used, there was minimal overlap between tumor and stroke LSM results, thereby substantiating our conclusion.

#### 4.4.2 Power across the brain

The power-problem in LSM studies is a heavily discussed topic (Ivanova et al., 2021, Sperber et al., 2018). Although our patient samples of stroke (*n* = 147) and tumor *(n =* 196) meet the general recommendations of 100–130 for LSM analyses, power does not only depend on the number of participants. Lesion distribution and volume also have a large effect upon the overall power of LSM studies. A sample with an average lesion volume of 3 cm^3^ will cover substantially less voxels than a sample with an average lesion volume of 10 cm^3^. Hence, the number of voxels with sufficient power for lesion-symptom analysis will be lower in study samples with smaller lesion sizes. As a result, the number of areas in which we had sufficient overlap to directly compare the LSM between the study samples was only 38 out of the 124 atlas areas. Unfortunately, post-hoc power analyses for multivariate inference cannot be computed, complicating the investigation of the exact effect of power on the multivariate LSM results. However, power analyses for the univariate LSM suggested that also in these areas of overlap, the number of voxels with adequate power to allow for replication was only a quarter to one-third. Overall, the number of voxels with adequate power was higher in tumor than in stroke, especially at a whole-brain level.

These power issues are not unique to our sample. The topography of damage and lesion volume of our stroke sample are comparable to the sample described by Corbetta and colleagues (*n* = 132) (Ramsey et al., 2017, Corbetta et al., 2018, Salvalaggio et al., 2020), although they included both hemorrhagic and ischemic strokes and relatively small lesions compared to our study. The authors mention the relatively small number of overlapping cortical lesions as one of the main limitations of the study. Even in a large-scale, multi-cohort LSM study (*n =* 2950) with high lesion coverage (average 2.7 cm^3^), some brain areas still achieved a maximum lesion overlap of five patients (Weaver et al., 2021), demonstrating the uneven lesion distribution that is inherent to the stroke population.

#### 4.4.3 Clinical representativeness

Our tumor sample included treatment-naïve diffuse glioma patients who underwent awake brain surgery. Consequently, the tumor population consisted of patients with different tumor grades and molecular markers. As edema, plasticity, lesion momentum and molecular markers are related to tumor grading, different tumor grades could be viewed as distinct (though related) etiologies. Additionally, previous research has indicated that certain molecular markers are independent determinants of cognitive functioning (van Kessel et al., 2022). Subgroup analysis stratified by tumor grade or IDH mutation, according to WHO 2021 classification (Louis et al., 2021), would be an interesting follow-up study. Stroke populations could also be further subdivided according to time since stroke onset (Karnath and Rennig, 2017). Current subgroup sample sizes were too small to perform this analysis with enough statistical power. Moreover, patients selected for awake surgery differ from patient under general anesthesia in various baseline characteristics (van Kessel et al., 2019). Additionally, the use of anti-epileptic medication and/or dexamethasone may add noise to the cognitive data of our tumor sample. Nevertheless, this will not lead to gross violations of the internal validity of the lesion-symptom associations. Stroke data was collected in a multi-site research project. Patients with larger infarction and typically also more severe clinical stroke symptoms, participate less frequently in research. Thus, LSM studies that include research participants may be systematically biased towards smaller strokes occurring in less eloquent areas and consequently less severe cognitive deficits. Although we have a large variation of stroke volume in our sample, the left-hemispheric strokes are considerably smaller than those in the right hemisphere. This is probably a direct result of the exclusion of patients who were too aphasic to participate. Still, the lesion-symptom associations that are found with research samples, such as our own, remain valid. Moreover, with a large enough sample size, small lesions will allow for better specificity in lesion-symptom associations than large lesions.

#### 4.4.4 Tumor delineation

We used the hyperintensities on the T2-Flair images to delineate tumor tissue. These hyperintensities are independent of the enhancement of the lesion, and thereby tumor grade, and thus form a widely usable representation of the extent of brain volume potentially hampered in its function. Our tumor lesion masks include tumor core, necrosis, and edema. We implicitly assumed that all visually abnormal tissue on the T2-image is damaged and functionally affected. However, the effect of edema and even tumor infiltration on function may vary within these areas and some function could be preserved. This is probably-one explanation for the inconsistent lesion-symptom associations across etiologies in this study. Future research may be aimed at determining the separate effects of edema, infiltration and enhancing tumor core on cognition.

#### 4.4.5 Lesion-network mapping

Recent insights from both structural and functional network studies, have shown the importance of connections between areas when investigating cognitive effects from lesions. The relevance of white matter damage to cognitive outcome is also supported by the current findings even when using an atlas with only 34 fiber bundles (de Haan and Karnath, 2017). Future work could use both stroke and tumor lesions as seeds in well-established structural or functional connectomes which can then be used as input for lesion network mapping analysis (Ferguson et al., 2019, Salvalaggio et al., 2020, Gleichgerrcht et al., 2017, Boes et al., 2015). Such studies could elucidate whether the cognitive effects of damage to critical white matter structures also differ between etiologies.

## 5 Conclusions

In this large-scale, direct comparison of LSM of patients with brain tumors versus ischemic stroke, we found substantial differences in lesion volume and topography between the groups. These differences partly drive the brain-behavior relationships that we found. In contrast to our expectations, the overlap of LSM in brain areas affected in both populations was minimal, though we did find white matter tracts involved in memory and semantic fluency performance across etiologies. Post-hoc analyses confirmed an interaction effect between lesion status and etiology for most brain areas with adequate coverage in both groups. Thus, we conclude that lesion-behavior associations as defined by LSM, are influenced by the etiology causing the lesion. Our findings cannot solely be explained by previous objections to the use of tumor patients in LSM. The pattern shown by tumor patients on the group level is consistent with localizations found in earlier studies using different techniques. In agreement with Shallice and Skrap (Shallice and Skrap, 2011), we argue that tumor series can be used to provide converging evidence about functional localization, next to evidence from other techniques such as functional imaging and direct electrocortical stimulation. In clinical terms, this study suggests that data from ischemic stroke patients have only limited value for the prediction of behavioral repercussions of specific lesions caused by primary brain tumors, and vice versa. Because of the lack of generalizability of findings across etiologies, we are cautious about grouping different etiologies in LSM, because results are easily driven by one population. Instead, we advocate to test predictions based on one etiology in a second patient population and explore divergent findings. It would be interesting, for example, to test to what extent divergent findings between tumor and stroke result from plasticity or preservation of function using cortical mapping observations during awake surgery.

## 6 Data availability statement

The results from the current study (lesion overlap maps, univariate and multivariate LSM, univariate statistical power maps) are available through the repository (van Grinsven, Eva; Smits, Anouk R. (2022), “Lesion-Symptom Mapping in Brain Tumor and Stroke Patients”, Mendeley Data, V1, https://doi.org/10.17632/k2847vw9gg.1, https://data.mendeley.com/datasets/k2847vw9gg). As the data is privacy-sensitive, individual patient data will be made available upon request. The requesting research should submit a formal project outline, and assessed whether this is in line with the original research protocol for the study. If not, additional approval from the local ethics committee may be necessary. If granted, a data sharing agreement will be signed by both parties. At all times the GDPR rules that are applicable at that moment will be followed when sharing the data. This, at minimum, indicates that MRI data will be defaced using the FreeSurfer MRI deface tool (https://surfer.nmr.mgh.harvard.edu/fswiki/mri_deface).

## CRediT authorship contribution statement

**E.E. van Grinsven:** Conceptualization, Data curation, Methodology, Formal analysis, Investigation, Visualization, Writing – original draft, Writing – review & editing. **A.R. Smits:** Conceptualization, Data curation, Methodology, Formal analysis, Investigation, Visualization, Writing – original draft, Writing – review & editing. **E. van Kessel:** Data curation, Investigation, Writing – review & editing. **M.A.H. Raemaekers:** Conceptualization, Methodology, Supervision, Software, Writing – review & editing. **E.H.F. de Haan:** Conceptualization, Funding acquisition, Methodology, Supervision, Writing – review & editing. **I.M.C. Huenges Wajer:** Writing – review & editing. **V.J. Ruijters:** Data curation, Investigation, Writing – review & editing. **M.E.P. Philippens:** Conceptualization, Supervision, Writing – review & editing. **J.J.C. Verhoeff:** Supervision, Writing – review & editing. **N.F. Ramsey:** Conceptualization, Methodology, Supervision, Resources, Writing – review & editing. **P.A.J.T. Robe:** Resources, Writing – review & editing. **T.J. Snijders:** Conceptualization, Supervision, Writing – review & editing. **M.J.E. van Zandvoort:** Conceptualization, Methodology, Supervision, Writing – review & editing.

## Declaration of Competing Interest

The authors declare that they have no known competing financial interests or personal relationships that could have appeared to influence the work reported in this paper.

## Data Availability

Data will be made available on request.
