# Supplementary material for: The impact of etiology in lesion-symptom mapping – A direct comparison between tumor and stroke
Source: Neuroimage Clin. 2022 Dec 24;37:103305. doi: 10.1016/j.nicl.2022.103305 (PMC9850191; doi:10.1016/j.nicl.2022.103305)
Supplement: Supplementary data 1 [file mmc1.docx]

Appendices belonging to the manuscript

**The impact of etiology in lesion-symptom mapping – A direct comparison between tumor and stroke**

# Appendix A

## In- and exclusion criteria

### **Tumor patients**

Patients who had any prior cancer treatment (i.e. operation [except biopsy before resection], chemotherapy, radiotherapy) were not included in the retrospective cohort. Additional inclusion criteria for the current study were availability of preoperative neurocognitive assessment, fluent in Dutch and a pre-operative T2 FLAIR MRI with slice thickness ≤5 mm in order to maintain adequate quality for lesion segmentation. No criterion was set for the amount of completed neurocognitive tasks. The tumor location on the T2 FLAIR had to be representative of the tumor at the time of the neurocognitive assessment as visible on other imaging modalities as determined by the neurologist (TS). Glioma patients with a history of neurological or psychiatric disease potentially interfering with the current neurocognitive performance or imaging were excluded for the current study.

### **Stroke patients**

Stroke patients were asked to participate if they were admitted to any of the following hospitals in the Netherlands between September 2015 and December 2019 in the Netherlands: Amsterdam University Medical Center (Amsterdam UMC), Radboud University Medical Center (Radboudumc) in Nijmegen, University Medical Center Groningen (UMCG), University Medical Center Utrecht (UMCU), Onze Lieve Vrouwe Gasthuis (OLVG), Maasziekenhuis Pantein, Rijnstate, Ommelander Ziekenhuis Groep, St. Antonius Ziekenhuis, and Diakonessenhuis. Patients were included for the current analyses if they were diagnosed with cerebral ischemic stroke made by a neurologist and fluent in Dutch. Exclusion criteria were 1) prior stroke based on imaging, 2) presence of another neurological, psychiatric or other diagnosis that may interfere with cognitive testing/imaging, 3) pre-existing cognitive decline (using a short informant-based questionnaire to asses change in cognitive functioning prior to stroke; cognitive decline was defined as a score >3.6 on the Dutch version of the Informant Questionnaire on Cognitive Decline in the Elderly [IQCODE]^1^).

## Neurocognitive assessment

All subjects underwent a neurocognitive assessment that included measures of language, attention, executive functions, memory, visuoconstruction and psychomotor speed. The neurocognitive assessment was administered via the standardized instructions and scoring was performed according to the standardized scoring criteria.  For tumor patients, the assessment was part of routine clinical care and the exact selection of tests was based on a standard protocol, which was tailored individually to patients’ complaints, tumor location and time constraints. Overall, most tumor patients completed the neurocognitive assessment within approximately two hours, including a break halfway through.  For the stroke patients a standard battery of tests was used. Overall, most stroke patients completed the neurocognitive assessment within approximately one and a half hours. **Supplementary table 1** shows the neuropsychological tasks and corresponding scores that were available for both the tumor and stroke sample.

For the lesion-symptom mapping analyses, we selected the Rey Auditory Verbal Learning Test (RAVLT, direct recall, delayed recall, and delayed recognition) and the verbal fluency test (Dutch versions of the Controlled Oral Word Association Test (COWAT) and Category Fluency (animal), both described in Bouma et al.^2^).  The RAVLT is a verbal learning and memory test that taps into multiple partly dissociated aspects of memory. Specifically, it assesses the ability to learn new information, consolidate it, reproduce it and recognize it after a delay period. During the task 15 unrelated nouns are read aloud on five consecutive trials. Each learning trials is followed by a free immediate recall test in which participants are asked to name as many words as they can remember. After a 20-minute delay, the participant is asked to recall the words from the list presented during the learning trials. Additionally, a list of 30 words is presented and the participant must identify the 15 previously presented words. From the RAVLT, we used the total number of words remembered on the five learning trials (immediate recall), the number of words remembered after the delay period (delayed recall) and number of correctly identified words in the recognition trial (delayed recognition).

The verbal fluency test is a short test of verbal functioning. More specifically, it assesses an individual’s ability to retrieve verbal information within restricted search parameters. The test is separated into a phonemic and semantic fluency part. In the phonemic fluency participants are given 60 seconds to generate as many unique words beginning with a single letter. Three trials, each with a different letter, are performed. In the semantic fluency test participants are given 60 seconds to generate unique words belonging to a certain semantic category, in this case animals. Both tasks require a complex interplay of a variety of cognitive functions including attention, vocabulary knowledge, retrieval of lexical and semantic knowledge and executive functions. The phonemic fluency test is thought to rely more heavily on executive control, while the semantic fluency test is more dependent on correct retrieval of semantic knowledge. For the letter fluency we used total number of correct words after three 60-seconds trials and for the semantic fluency the number of correct words within the category.

**Supplementary Table 1 Neuropsychological tasks and corresponding scores.**

| **Neuropsychological task** | **Cognitive domain** | **Raw score** |
| --- | --- | --- |
| Boston Naming Test^a^ | Naming ability | Total correct |
| Letter fluency^b^ | Verbal fluency | Total amount of correct, unique words over three 60-seconds trials |
| Semantic fluency^c^ | Verbal fluency | Total amount of correct, unique words within 60 seconds |
| RAVLT - Dutch version^d^ | Verbal memory | Immediate recall (trial 1-5) |
|  |  | Delayed recall score |
|  |  | Delayed recognition score |
| ROCFT^e^ | Visuoconstructive abilities | Direct copy |
| WAIS-III or WAIS-IV Digit Span^f^ | Attention Span | Forward span |
|  | Working Memory | Backward span |
| Trail Making Test^g^ | Psychomotor speed | Time to complete part A |
|  | Switching | Ratio score (B/A) |

Abbreviations: RAVLT, Rey Auditory Verbal Learning Test; ROCFT; Rey-Osterieth Complex Figure Test; WAIS, Wechsler Adult Intelligence Scale;

^a^Boston Naming Task, Heesbeen, Van Loon-Vervoorn, 2001.

^b^Phonologic Verbal Fluency Test (Lexical Fluency) (Harrison, Buxton,

Husain, Wise, 2010; Schmand, Groenink, Van Den Dungen, 2008).

^c^Semantic Verbal Fluency Test, Harrison et al, 2010 (Gerritsen et al., Nederlands Instituut van Psychologen, 2012).

^d^15 Words Test (15WT) (Saan, Deelman, 1986).

^e^Berry, Carpenter, 1992; Spreen, Strauss, 1998

^f^Wechsler Adult Intelligence Scale Third Edition Digit Span (WAIS-III) (WAIS-III Administration and scoring manual, 1997), Wechsler Adult Intelligence Scale Fourth Edition Digit Span (WAIS-IV) (WAIS-IV-NL Technische handleiding, 2013).

^g^ Giovagnoli, Del Pesce, Mascheroni, Simoncelli, Laiacona, Capitani, 1996 (Drane, Yuspeh, Huthwaite, & Klinger, 2002).

## Supplementary references

1. de Jonghe JF, Schmand B, Ooms ME, M. W. Ribbe. Abbreviated form of the Informant Questionnaire on cognitive decline in the elderly. *Tijdschr Gerontol Geriatr*. 1997;28(5):224-229.

2. Bouma A, Mulder J, Lindeboom J SB. *Handboek Neuropsychologische Diagnostiek*. Pearson; 2012.

# Appendix B – Lesion volume

**Supplementary Table 2. Spearman Rho correlation values and *p* values for the association between lesion volume and performance on cognitive tasks**

|  | **Glioma** | **Stroke** | **Total** |
| --- | --- | --- | --- |
| Boston Naming Test | ***r* = -.288**  ***p* < .001** | *r* = -.099  *p* = .163 | ***r* = -.362**  ***p* < .001** |
| Phonological fluency | ***r* = -.363**  ***p* < .001** | *r* = -0.49  *p* = .339 | ***r* = -.126**  ***p* = .045** |
| Semantic fluency | ***r* = -.341**  ***p* < .001** | *r* = -.038  *p* =.336 | *r* = -.095  *p* = .059 |
| RAVLT – direct recall | ***r* = -.272**  ***p* < .001** | *r* = -.021  *p* = .409 | *r* = -.078  *p* = .088 |
| RAVLT – delayed recall | ***r* = -.238**  ***p* < .001** | *r* = .031  *p* = .366 | ***r* = -.101**  ***p* = .041** |
| RAVLT – delayed recognition | ***r* = -.254**  ***p* < .001** | *r* = -.143  *p* = .057 | ***r* = -.177**  ***p* = .001** |
| ROCFT direct copy | ***r* = -.198**  ***p* = .005** | *r* = -.135  *p* = .306 | *r* = -.032  *p* < .310 |
| WAIS digit span forward | ***r* = -.215**  ***p* = .001** | *r* = -.137  *p* = .057 | ***r* = -.240**  ***p* < .001** |
| WAIS digit span backward | ***r* = -.261**  ***p* < .001** | *r* = -.100  *p* = .125 | ***r* = -.435**  ***p* < .001** |
| TMT A | ***r* = -.276**  ***p* < .001** | ***r* = -.210**  ***p* = .009** | ***r* = -.119**  ***p* = .019** |
| TMT B ratio | ***r* = -.180**  ***p* = .010** | *r* = .082  *p* = .182 | *r* = -.087  *p* = .068 |
| Mean overall Z score | ***r* = -.415**  ***p* < .001** | *r* = -.139  *p* = .051 | ***r* = -.172**  ***p* = .001** |

**
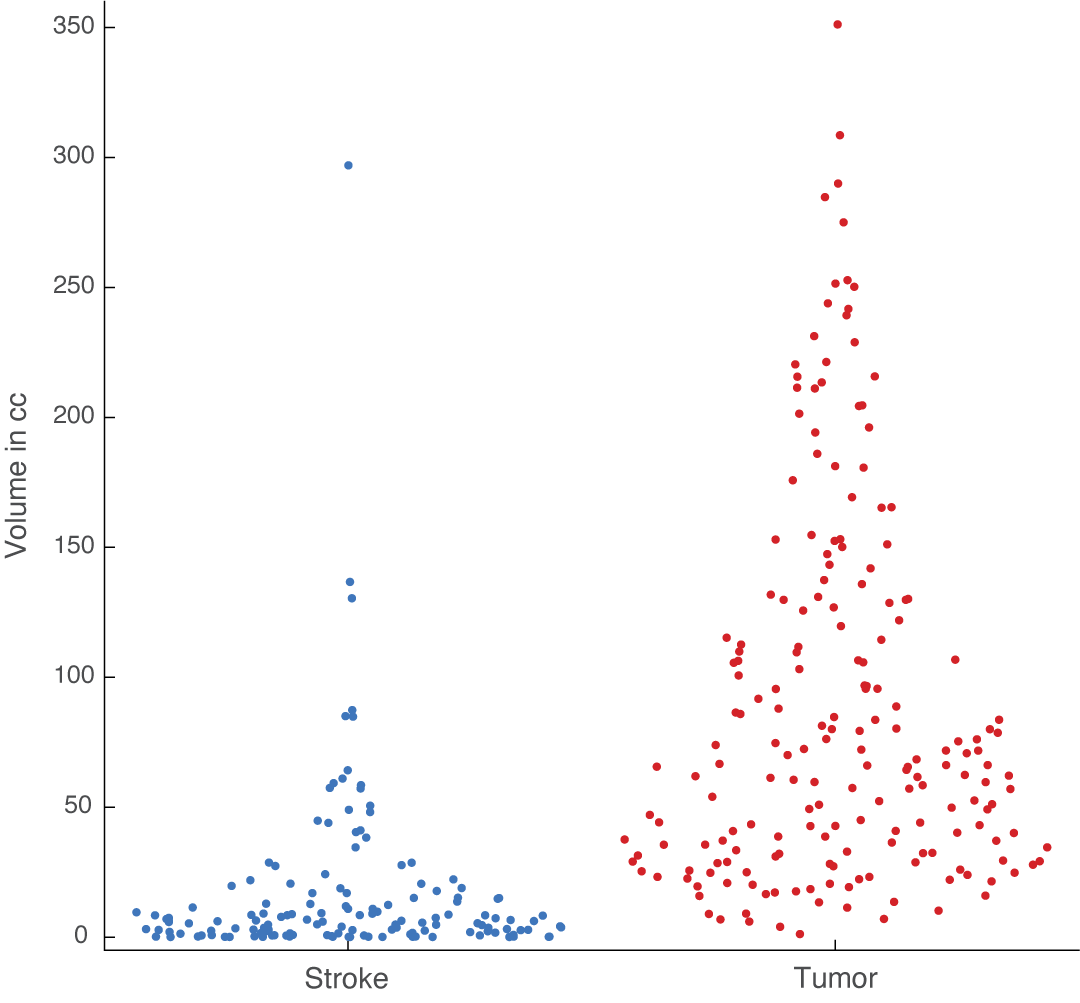
**

**Supplementary Figure 1. Swarm chart of lesion volumes for the tumor and stroke group.** Each dot represents the lesion volume of one patient with stroke patients represented by blue and tumor patients by red dots.

**Supplementary Table 3. Demographic and cognitive characteristics of the stroke group, subdivided by lesion volume.**

|  | **Stroke** | |
| --- | --- | --- |
| Lesion volume | ≤3 cc | >3 cc |
| N | 54 | 93 |
| Sex (%) |  |  |
| Male | 39 (72.2) | 60 (64.5) |
| Female | 15 (27.8) | 33 (35.5) |
| Mean age (SD) | 57.3 | 57.5 |
| Range | 19-81 | 20-82 |
| Lesion location (%) |  |  |
| Left | 27 (50.0) | 39 (41.9) |
| Right | 20 (37.0) | 46 (49.5) |
| Bilateral | 7 (13.0) | 8 (8.6) |
| Cognitive performance ≤-1.5 (%)* |  |  |
| Boston Naming Test | 2 (3.7) | 0 (0.0) |
| Digit Span Forward | 5 (9.4) | 10 (11.4) |
| Digit Span Backward** | 5 (9.4) | 21 (23.9) |
| RAVLT – Direct recall | 5 (9.8) | 12 (14.6) |
| RAVLT – Delayed recall | 9 (17.6) | 13 (16.0) |
| RAVLT – Delayed recognition | 6 (11.8) | 8 (9.9) |
| ROCFT – Copy | 11 (20.4) | 11 (23.4) |
| Semantic fluency** | 1 (2.1) | 13 (15.5) |
| Letter fluency | 6 (11.1) | 10 (19.6) |
| TMT A | 2 (3.8) | 8 (9.8) |
| TMT B/A ratio | 2 (4.0) | 5 (6.2) |

* valid percentage is shown thus based on N of patients with a specific test score

** significant difference (p < .05) between groups

# Appendix C – Combined SVR-LSM

We combined the data of both groups and performed the SVR-LSM analyses for each cognitive task using etiology (tumor or stroke) as a covariate on both the behavioral scores and lesion data. In areas where both groups have adequate coverage, and thus etiology can be used as covariate, this analysis allows investigation of the relation between lesion location and cognitive performance, irrespective of etiology. It is important to note that this analysis is still subject to differences in sample size between the groups and results can still be driven by one etiology simply because its sample size is significantly larger.

## Direct recall verbal memory (Supplementary Figure 2)

When both groups were combined for the SVR-LSM analyses, lesions in the left ILF (most voxels with peak significance; 11.8%) were most strongly associated with performance on the RAVLT direct recall. Lesions in the left optic radiation (17.3%) and posterior segment (12.2%) were also associated with worse task performance. Additionally multiple grey areas were associated with direct recall performance, among which the left hippocampus (38.2%), the thalamus (15.5%), the parahippocampal gyrus (14.8%), the inferior temporal gyrus (14.4%) and the caudate nucleus (13.4%). When comparing these results to the etiology-specific lesion-symptom maps, largely overlapping voxels were found (**Supplementary Figure 2** and **Supplementary** **Table 5**). Nevertheless, some brain areas were only significant in the combined maps (left inferior temporal gyrus) and others only in the etiology specific maps (left uncinate fasciculus, lingual gyrus).


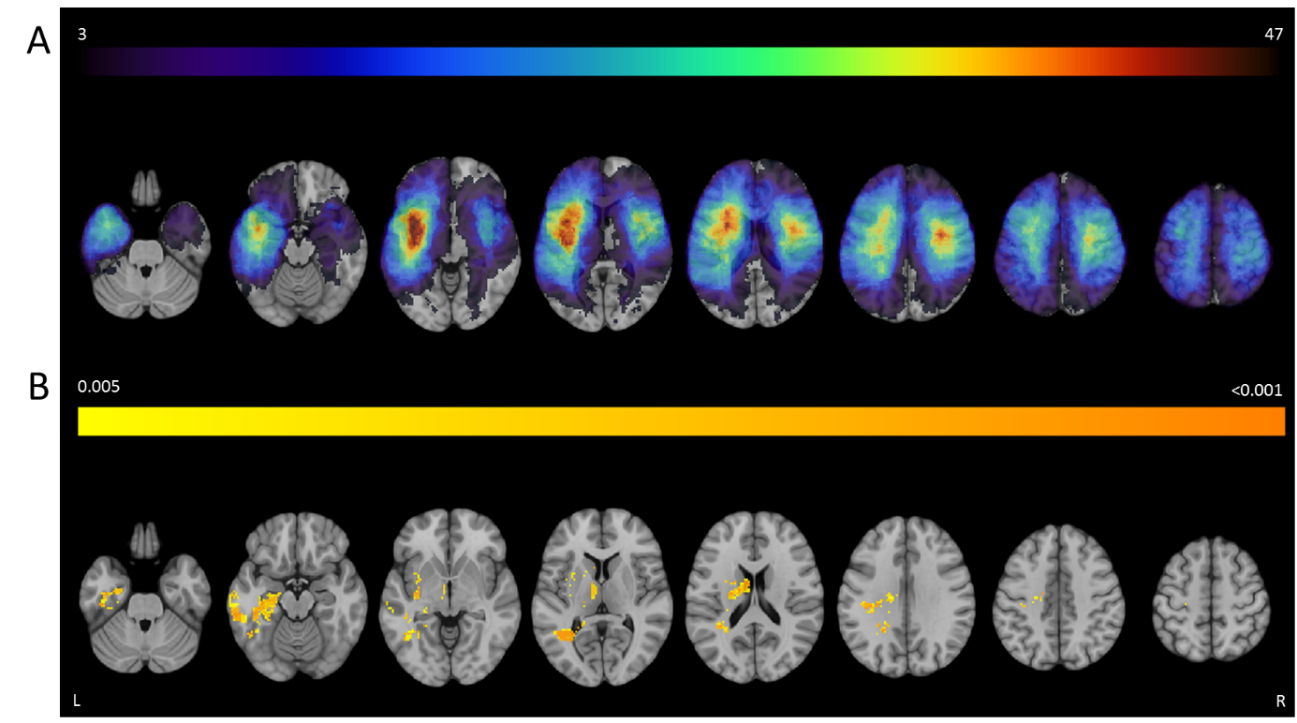


**Supplementary Figure 2. SVR-LSM results for the RAVLT direct recall for both groups combined when etiology was added as covariate.** Panel A shows the lesion overlap for this task when lesions from both groups are combined. The color bar indicates the number of patients with overlapping lesions. Panel B shows the voxels that were significantly associated with worse performance (yellow). The yellow color indicates the p-value for each voxel.

## Delayed recall verbal memory (Supplementary Figure 3)

In the combined SVR-LSM analysis, lesions in the left ILF (most voxels with peak significance; 26.2%), optic radiation (29.2%), posterior segment (14.7%) and IFOF (12.3%) were strongly associated with task performance. Significant voxels extended into grey matter areas, including the left hippocampus (39.2%), parahippocampal gyrus (18.1 %), inferior temporal gyrus (12.3%). Significant areas largely overlapped with the areas found when the analysis was run for the tumor and stroke group separately (**Supplementary** **Figure 3** and **Supplementary** **Table 5**). However, while lesions in the left putamen were highly associated with worse task performance in the stroke group, this area was not significant in the combined group SVR-LSM analysis.


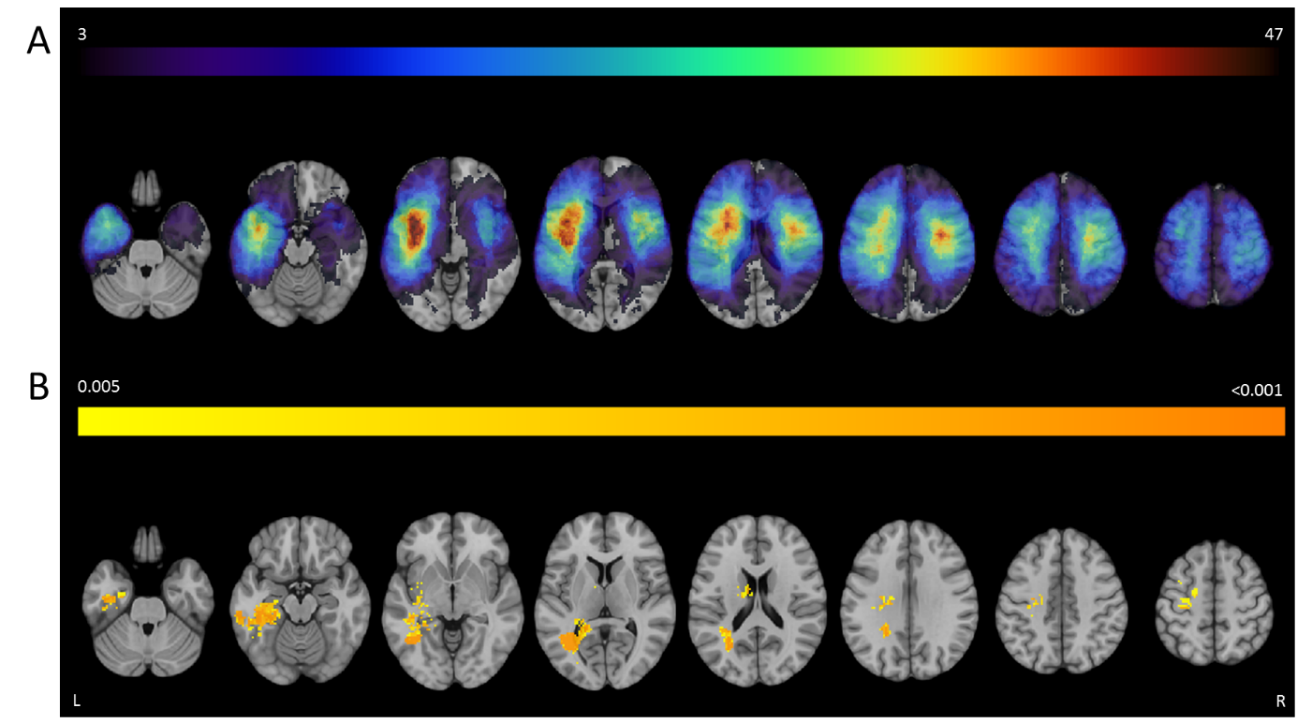


**Supplementary Figure 3. SVR-LSM results for the RAVLT delayed recall for both groups combined when etiology was added as covariate.** Panel A shows the lesion overlap for this task when lesions from both groups are combined. The color bar indicates the number of patients with overlapping lesions. Panel B shows the voxels that were significantly associated with worse performance (yellow). The yellow color indicates the p-value for each voxel.

## Delayed recognition verbal memory (Supplementary Figure 4)

Multiple brain areas in of the left hemisphere were significantly associated with task performance in the combined group analysis. Significant grey matter brain areas included the middle temporal gyrus (most voxels with peak significance; 38.1%), the hippocampus (50.0%), the inferior temporal gyrus (32.9%), the parahippocampal gyrus (14.9%), the fusiform gyrus (11.6%) and the superior temporal gyrus (11.2%). Significant voxels extended into white matter areas like the ILF (58.9%), posterior segment (31.4%), optic radiation (31.1%) and the IFOF (14.5%). Although most brain areas overlapped with those found in the etiology specific lesion-symptom maps some brain areas were found in the combined group analysis that were not found when analyzing the groups separately, like the left superior temporal gyrus (**Supplementary Figure 4** and **Supplementary** **Table 5**). Additionally, all right-sided brain areas that were involved in task performance in the stroke group (inferior and middle frontal gyrus), were not indicated by the combined group analysis.


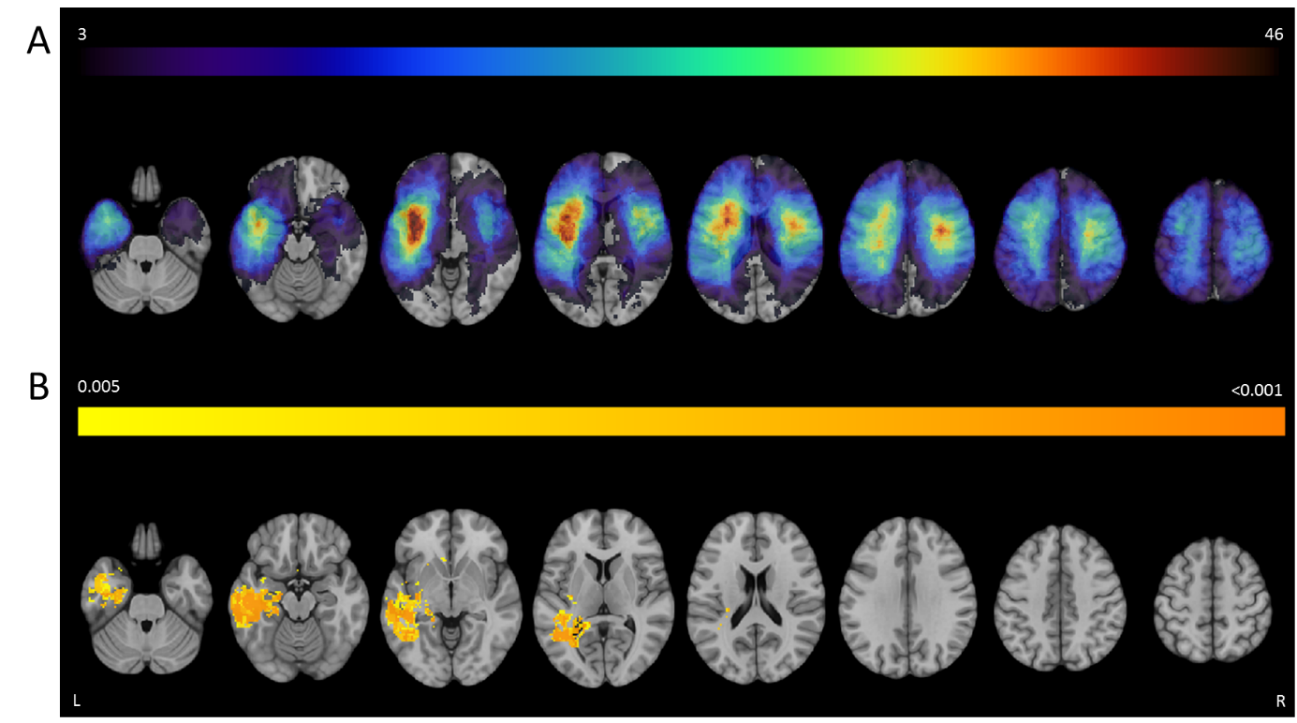


**Supplementary Figure 4. SVR-LSM results for the RAVLT delayed recognition for both groups combined when etiology was added as covariate.** Panel A shows the lesion overlap for this task when lesions from both groups are combined. The color bar indicates the number of patients with overlapping lesions. Panel B shows the voxels that were significantly associated with worse performance (yellow). The yellow color indicates the p-value for each voxel.

## Letter fluency (Supplementary Figure 5)

Lesions within the left insula (most voxels with peak significance, 22.7%) were most strongly associated with worse letter fluency performance when the groups were combined. Additionally, the left putamen (35.1%), inferior frontal gyrus opercular (24.4%), caudate nucleus (15.9%) and long segment (12.1%) were significantly involved in the task performance. In general, there was good consensus with brain areas found in the etiology specific and combined lesion-symptom maps (**Supplementary Figure 5** and **Supplementary** **Table 5**).


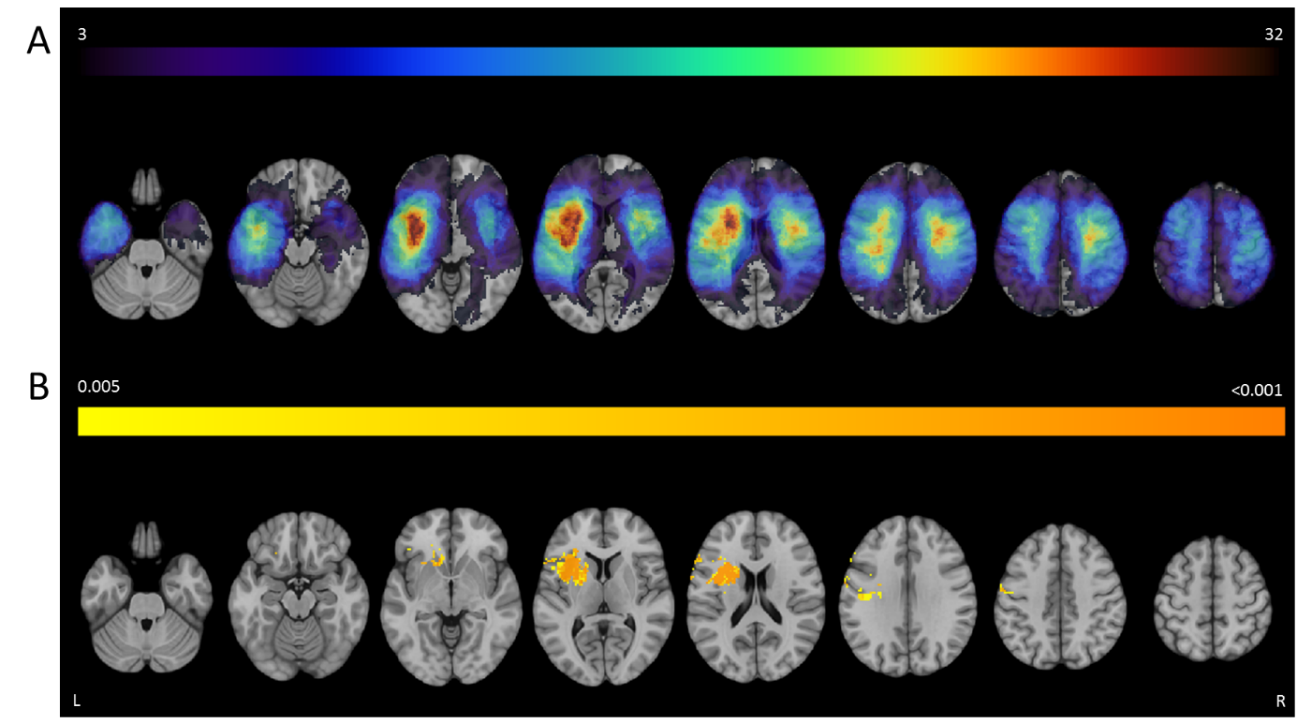


**Supplementary Figure 5. SVR-LSM results for the letter fluency for both groups combined when etiology was added as covariate.** Panel A shows the lesion overlap for this task when lesions from both groups are combined. The color bar indicates the number of patients with overlapping lesions. Panel B shows the voxels that were significantly associated with worse performance (yellow). The yellow color indicates the p-value for each voxel.

## Semantic fluency (Supplementary Figure 6)

The combined SVR-LSM analyses indicated lesions in the left corticospinal tract (most voxels with peak significance; 14.4%) to be most strongly related to worse semantic fluency performance. Significant voxels extended into grey matter areas, including the caudate nucleus (23.1%) and the precentral gyrus (21.5%). While at large the brain areas found in the etiology specific lesions-symptom maps were also found in the combined lesion-symptom maps, some differences were apparent. For example, the caudate nucleus was only significant when both groups were combined and the left superior dorsolateral frontal gyrus and rolandic operculum were only found in the separate tumor and stroke analyses, respectively. (**Supplementary Figure 6** and **Supplementary** **Table 5**).


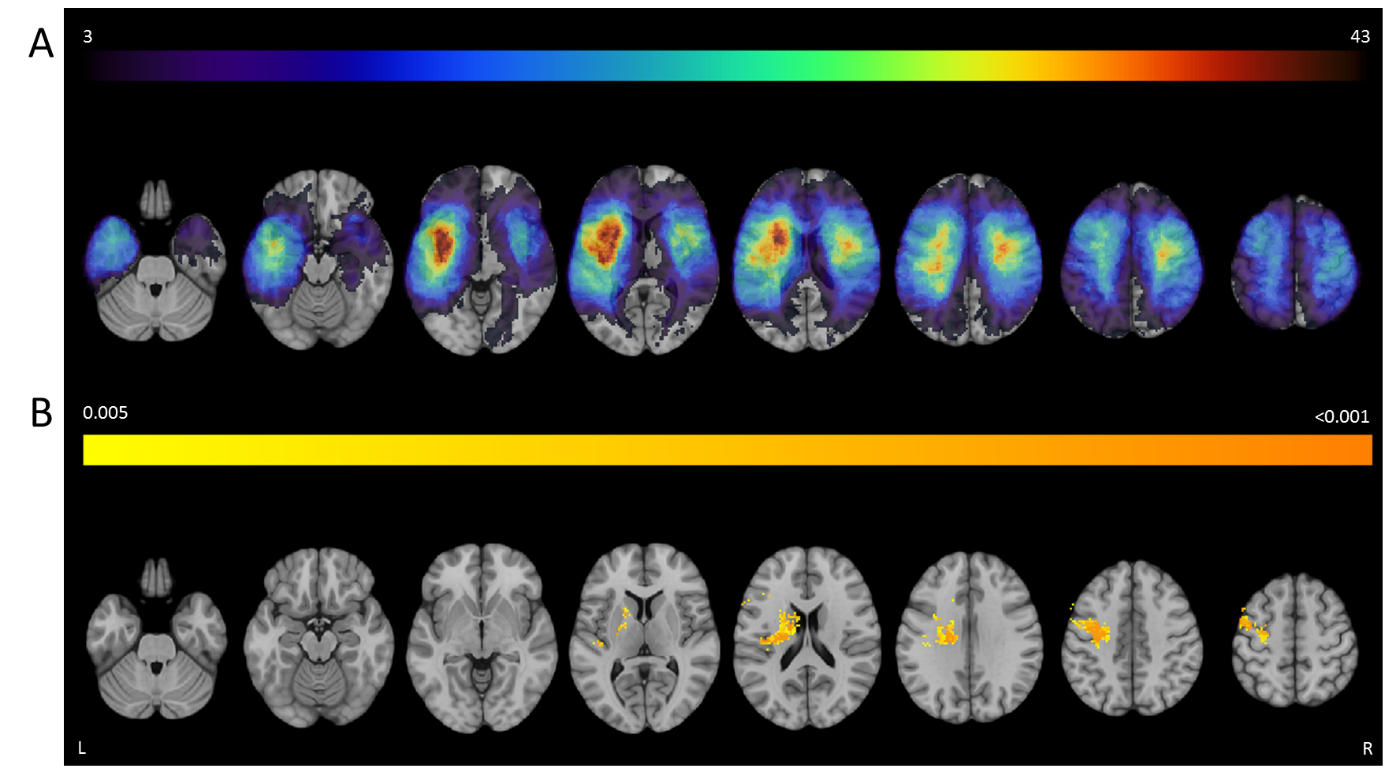


**Supplementary Figure 6. SVR-LSM results for the semantic fluency for both groups combined when etiology was added as covariate.** Panel A shows the lesion overlap for this task when lesions from both groups are combined. The color bar indicates the number of patients with overlapping lesions. Panel B shows the voxels that were significantly associated with worse performance (yellow). The yellow color indicates the p-value for each voxel.

**Supplementary Table 4 Average lesion frequency, including all tumor and stroke patients.** Grey matter areas are in reference to the AAL atlas, white matter areas in reference to the CAT atlas. Only those areas are shown in which at least 3 patients had a lesion. The average overlap is the percentage of patients with a lesion in that area compared to the total group.

| Area name | | % overlap of total | |  | Area name | | % overlap of total | |
| --- | --- | --- | --- | --- | --- | --- | --- | --- |
|  |  | Tumor | Stroke |  |  |  | Tumor | Stroke |
| *Grey matter areas* | |  |  |  |  | |  |  |
| Precentral gyrus | L | 19.39 | 4.08 |  | Superior occipital lobe | L | 7.14 |  |
|  | R | 17.35 | 8.16 |  |  | R | 5.10 | 4.76 |
| Superior frontal gyrus, dorsolateral | L | 15.82 | 2.04 |  | Middle occipital lobe | L | 10.20 | 2.04 |
|  | R | 11.73 | 5.44 |  |  | R | 5.10 | 3.40 |
| Superior frontal gyrus, orbital | L | 16.84 | 2.72 |  | Inferior occipital lobe | L | 5.61 |  |
|  | R | 3.57 | 2.04 |  |  | R | 1.53 | 3.40 |
| Middle frontal gyrus | L | 16.33 | 2.72 |  | Fusiform gyrus | L | 16.33 |  |
|  | R | 12.76 | 7.48 |  |  | R | 3.06 | 4.08 |
| Middle frontal gyrus, orbital | L | 9.18 |  |  | Postcentral gyrus | L | 18.37 | 4.08 |
|  | R | 2.55 | 2.04 |  |  | R | 17.35 | 6.12 |
| Inferior frontal gyrus, opercular | L | 23.47 | 5.44 |  | Superior parietal gyrus | L | 11.73 | 2.04 |
|  | R | 13.78 | 10.88 |  |  | R | 11.22 | 4.76 |
| Inferior frontal gyrus, triangular | L | 20.92 | 3.40 |  | Inferior parietal gyrus | L | 15.82 | 3.40 |
|  | R | 10.20 | 7.48 |  |  | R | 13.27 | 4.76 |
| Inferior frontal gyrus, orbital | L | 18.37 | 2.72 |  | Supramarginal gyrus | L | 15.31 | 3.40 |
|  | R | 5.10 | 7.48 |  |  | R | 15.31 | 4.08 |
| Rolandic operculum | L | 22.96 | 6.80 |  | Angular gyrus | L | 12.76 | 3.40 |
|  | R | 15.82 | 10.20 |  |  | R | 9.18 | 4.08 |
| Supplementary motor area | L | 12.76 |  |  | Precuneus | L | 9.69 | 2.04 |
|  | R | 8.16 |  |  |  | R | 8.67 | 2.72 |
| Olfactory cortex | L | 15.82 |  |  | Paracentral lobule | L | 9.69 |  |
|  | R | 5.10 | 4.08 |  |  | R | 8.16 |  |
| Superior frontal gyrus, medial | L | 11.73 |  |  | Caudate nucleus | L | 18.88 | 7.48 |
|  | R | 6.63 |  |  |  | R | 9.69 | 8.84 |
| Superior frontal gyrus, medial orbital | L | 7.14 |  |  | Putamen | L | 21.94 | 9.52 |
|  | R | 3.06 |  |  |  | R | 10.71 | 11.56 |
| Gyrus rectus | L | 13.27 |  |  | Pallidum | L | 18.37 | 7.48 |
|  | R | 3.57 |  |  |  | R | 6.63 | 7.48 |
| Insula | L | 24.49 | 8.16 |  | Thalamus | L | 11.73 | 2.04 |
|  | R | 14.80 | 11.56 |  |  | R | 6.12 |  |
| Cingulate gyrus, anterior part | L | 12.76 |  |  | Heschl gyrus | L | 21.43 | 6.80 |
|  | R | 9.18 |  |  |  | R | 13.27 | 4.76 |
| Cingulate gyrus, mid part | L | 13.27 |  |  | Superior temporal gyrus | L | 22.96 | 4.76 |
|  | R | 9.18 |  |  |  | R | 11.73 | 4.76 |
| Cingulate gyrus, posterior part | L | 3.06 |  |  | Temporal pole: superior temporal gyrus | L | 21.94 | 2.72 |
|  | R | 2.04 |  |  |  | R | 10.20 | 4.76 |
| Hippocampus | L | 19.39 | 2.04 |  | Middle temporal gyrus | L | 19.39 | 2.72 |
|  | R | 4.59 | 2.04 |  |  | R | 6.12 | 4.08 |
| ParaHippocampal gyrus | L | 15.31 | 2.04 |  | Temporal pole: middle temporal gyrus | L | 14.29 | 2.04 |
|  | R | 4.08 | 4.08 |  |  | R | 4.59 | 3.40 |
| Amygdala | L | 15.31 |  |  | Inferior temporal gyrus | L | 16.33 | 2.04 |
|  | R | 3.57 |  |  |  | R | 3.06 | 2.04 |
| Calcarine fissure and surrounding cortex | L | 7.14 | 2.72 |  | Cerebellum crus 1 | L | 3.06 |  |
|  | R | 3.57 | 7.48 |  | Cerebellum crus 2 | L |  | 2.04 |
| Cuneus | L | 6.12 | 2.04 |  | Cerebellum 4 5 | L | 3.57 |  |
|  | R | 3.57 | 3.40 |  |  | R | 1.53 |  |
| Lingual gyrus | L | 10.20 | 2.72 |  | Cerebellum 6 | L | 4.08 |  |
|  | R |  | 4.76 |  |  | R |  | 2.04 |
|  |  |  |  |  |  |  |  |  |
| *White matter areas* |  |  |  |  |  |  |  |  |
| Anterior Commissure | L | 21.43 | 6.12 |  | Inferior Longitudinal Fasciculus | L | 23.98 | 3.40 |
|  | R | 6.63 | 4.76 |  |  | R | 8.16 | 3.40 |
| Anterior Segment | L | 20.92 | 4.76 |  | Inferior Fronto-Occipital Fasciculus | L | 23.98 | 7.48 |
|  | R | 18.88 | 9.52 |  |  | R | 10.20 | 9.52 |
| Arcuate Fasciculus | L | 20.92 | 5.44 |  | Internal Capsule | L | 22.96 | 8.16 |
|  | R | 19.39 | 9.52 |  |  | R | 15.82 | 10.20 |
| Cingulum | L | 17.86 | 2.72 |  | Long Segment | L | 21.43 | 5.44 |
|  | R | 12.76 | 5.44 |  |  | R | 16.84 | 6.12 |
| Corpus Callosum | L | 20.41 | 4.08 |  | Optic Radiation | L | 22.96 | 6.80 |
|  | R | 13.27 | 8.16 |  |  | R | 10.20 | 4.76 |
| Cortico-ponto-cerebellar tract | L | 19.90 | 6.80 |  | Posterior Segment | L | 18.88 | 2.04 |
|  | R | 15.82 | 7.48 |  |  | R | 10.20 | 3.40 |
| Corticospinal tract | L | 22.45 | 9.52 |  | Superior Cerebellar Peduncule | L | 5.10 |  |
|  | R | 17.86 | 10.20 |  |  | R | 3.06 |  |
| Fornix | L | 23.98 | 2.72 |  | Uncinate Fasciculus | L | 22.96 | 7.48 |
|  | R | 11.22 | 2.72 |  |  | R | 7.14 | 9.52 |

**Supplementary Table 5 Detailed descriptions of the anatomical location of significant voxels identified by the SVR-LSM analyses when the tumor and stroke group were combined.** Numbers represent the percentage of tested voxels that were significant for that atlas in SVR-LSM analyses. Starred atlas areas contain the peak z-values in most voxels for each cognitive task. If a task was found to be associated with more than 5 atlas areas, only tested areas containing at least 5% significant voxels are reported. Smaller clusters are included if they contained peak z-values for that task. Grey matter areas are listed above the dotted horizontal line, white matter areas below. All anatomical areas are defined based on the AALCAT atlas. Abbreviations: Hem, hemisphere; L, left; R, right.

| Anatomical location | Hem | Direct recall | Delayed recall | Recognition | Letter fluency | Semantic fluency |
| --- | --- | --- | --- | --- | --- | --- |
| Precentral gyrus | L |  | 5.6 |  |  | 21.5 |
| Inferior frontal gyrus. opercular | L |  |  |  | 24.4 |  |
| Inferior frontal gyrus. triangular | L |  |  |  | 9.0 |  |
| Rolandic operculum | L |  |  |  | 8.0 |  |
| Insula | L |  |  |  | 22.7* |  |
| Hippocampus | L | 38.2 | 39.2 | 50.0 |  |  |
| ParaHippocampal gyrus | L | 14.8 | 18.1 | 14.9 |  |  |
| Fusiform gyrus | L | 8.3 | 12.9 | 11.6 |  |  |
| Caudate nucleus | L | 13.4 |  |  | 15.9 | 23.1 |
| Putamen | L | 6.3 |  |  | 35.1 |  |
| Thalamus | L | 15.5 |  |  |  |  |
| Heschl gyrus | L |  |  |  |  | 6.2 |
| Superior temporal gyrus | L |  |  | 11.2 |  |  |
| Middle temporal gyrus | L | 6.1 | 5.3 | 38.1* |  |  |
| Inferior temporal gyrus | L | 14.4 | 17.6 | 32.9 |  |  |
| Anterior segment | L |  |  |  | 5.8 | 8.7 |
| Arcuate fasciculus | L | 6.1 |  |  |  | 5.9 |
| Cingulum | L |  | 7.3 | 7.0 |  |  |
| Corpus callosum | L |  | 6.0 |  |  |  |
| Corticospinal tract | L | 7.9 | 8.0 |  | 8.1 | 14.4* |
| Fornix | L | 7.5 | 5.4 | 6.6 |  |  |
| Inferior Longitudinal Fasciculus | L | 11.8* | 26.2* | 58.9 |  |  |
| Inferior Fronto-Occipital Fasciculus | L | 7.2 | 12.3 | 14.5 | 8.0 |  |
| Internal capsule | L | 6.3 | 6.9 |  | 5.4 | 7.2 |
| Long segment | L |  |  |  | 12.1 | 8.3 |
| Optic Radiation | L | 17.3 | 29.2 | 31.1 |  |  |
| Posterior Segment | L | 12.2 | 14.7 | 31.4 |  |  |
| Uncinate Fasciculus | L |  |  | 5.4 | 5.3 |  |

# Appendix D – Univariate vs Multivariate LSM

We performed univariate LSM to corroborate results found in the multivariate analyses. The Supplementary Figures 7-10 visualize the LSM results that indicated a significant association between lesioned voxels and worse cognitive performance on both the univariate and multivariate analyses.

**
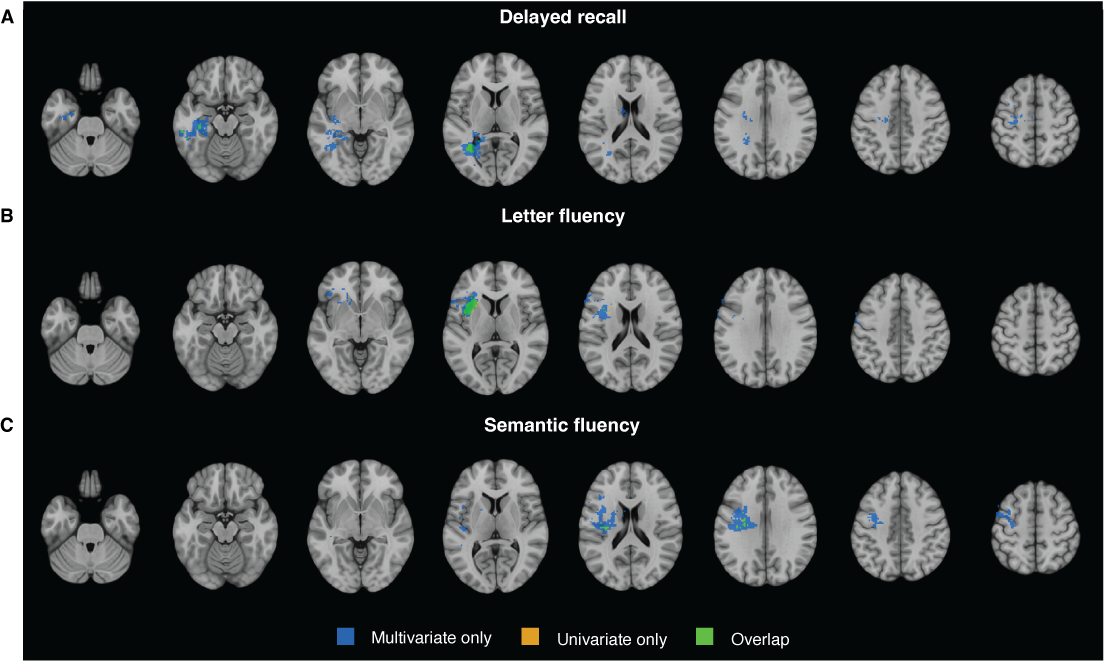
Supplementary Figure 7. Lesion-symptom mapping results with SVR-LSM or VLSM for the RAVLT delayed recall (A), letter fluency (B) and semantic fluency (C) for the tumor group.** Voxels significantly associated with worse performance are color-coded indicating voxels only found in multivariate analyses in blue, only found in univariate analyses in orange and voxels found by both analyses in green.


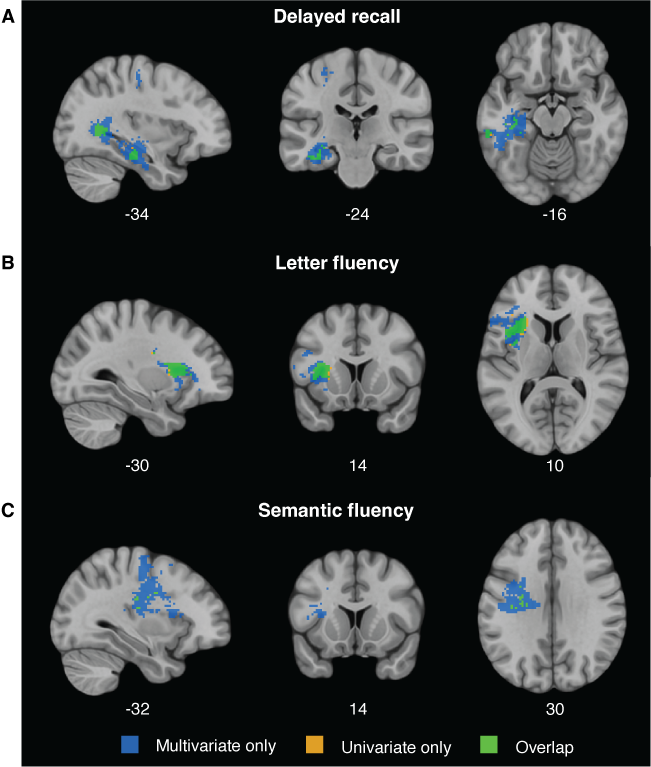


**Supplementary Figure 8. Lesion-symptom mapping results with SVR-LSM or VLSM for the RAVLT delayed recall (A), letter fluency (B) and semantic fluency (C) for the tumor group.** Voxels significantly associated with worse performance are color-coded indicating voxels only found in multivariate analyses in blue, only found in univariate analyses in orange and voxels found by both analyses in green. Numbers underneath the slices indicate the slice position in MNI space.


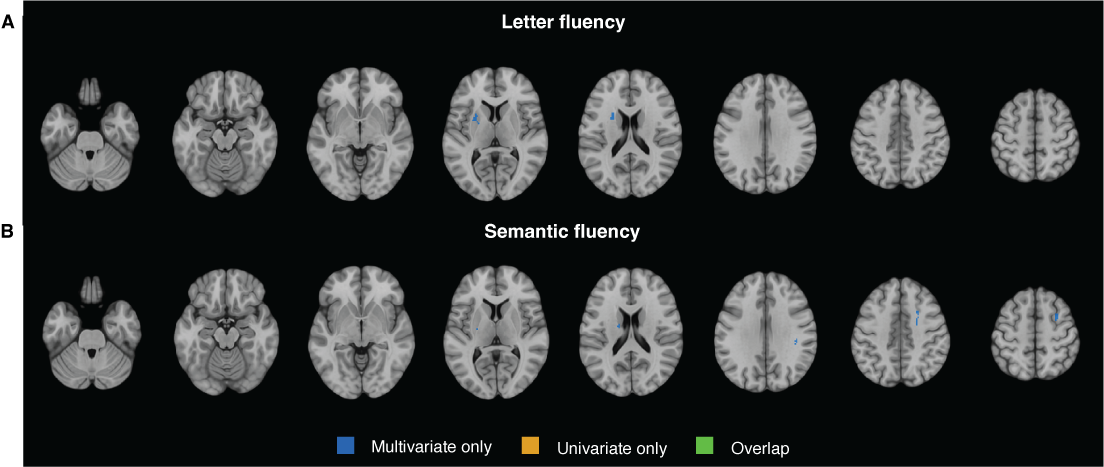


**Supplementary Figure 9. Lesion-symptom mapping results with SVR-LSM or VLSM for the RAVLT delayed recall (A), letter fluency (B) and semantic fluency (C) for the stroke group.** Voxels significantly associated with worse performance are color-coded indicating voxels only found in multivariate analyses in blue, only found in univariate analyses in orange and voxels found by both analyses in green.


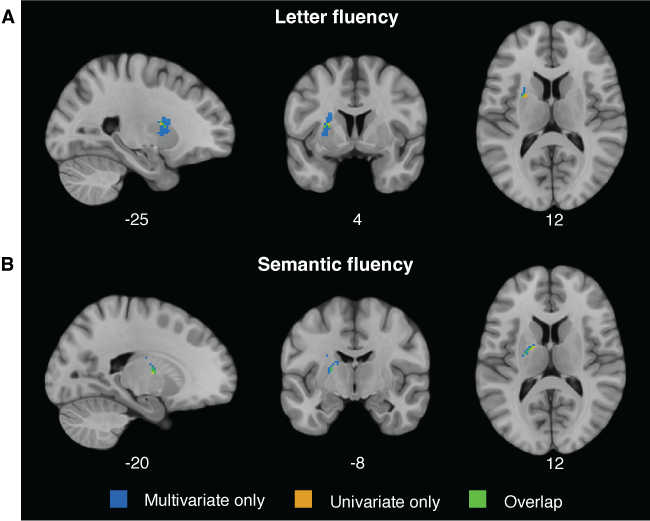


**Supplementary Figure 10. Lesion-symptom mapping results with SVR-LSM or VLSM for the RAVLT delayed recall (A), letter fluency (B) and semantic fluency (C) for the stroke group.** Voxels significantly associated with worse performance are color-coded indicating voxels only found in multivariate analyses in blue, only found in univariate analyses in orange and voxels found by both analyses in green. Numbers underneath the slices indicate the slice position in MNI space.

# Appendix E – Univariate LSM Powermaps


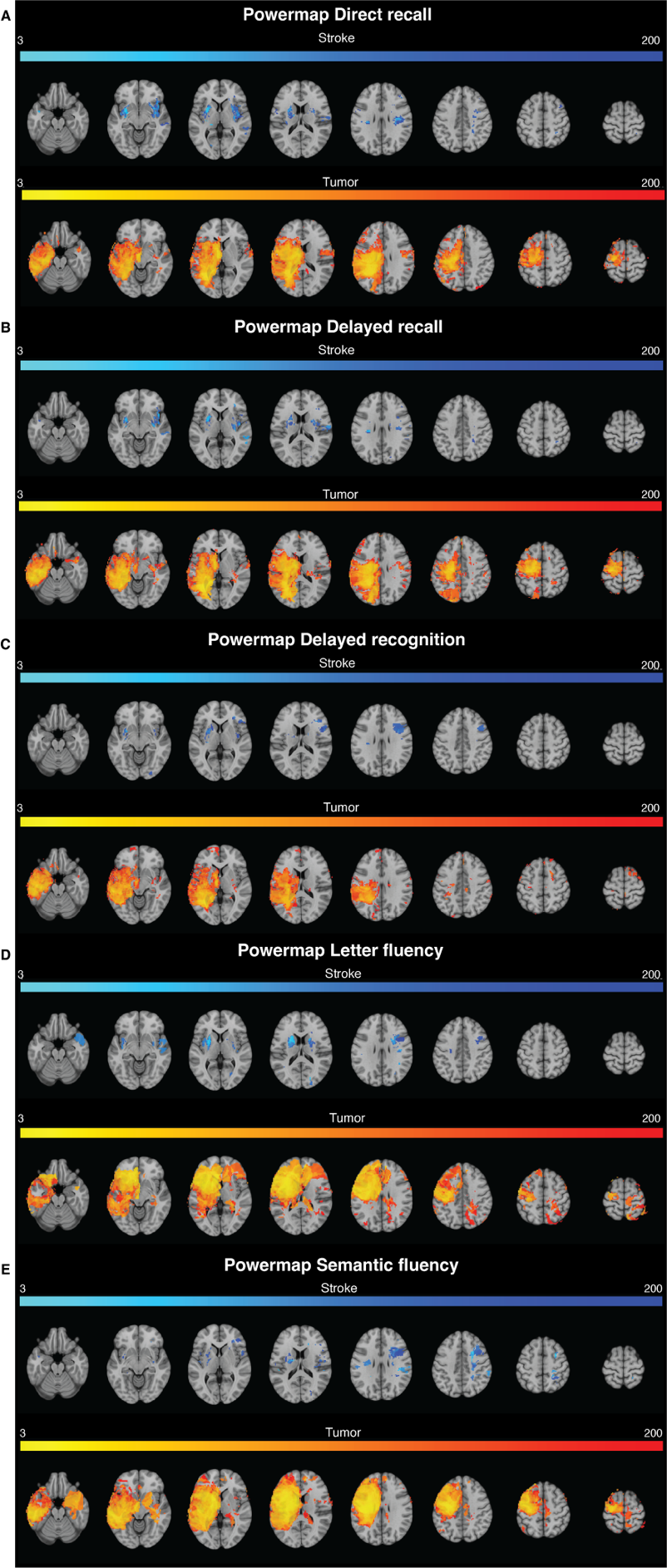


**Supplementary Figure 11. VLSM power maps representing the number of patients that would be needed to replicate the results in 60% of the studies, shown separately for the stroke (blue-light blye) and tumor (yellow-red) group for the RAVLT Direct recall (A), Delayed recall (B), Delayed recognition (C), Letter fluency (D) and Semantic fluency (D).** Statics are superimposed on the MNI brain.
